# Comparative Proteomic Analysis Reveals the Effect of Selenoprotein W Deficiency on Oligodendrogenesis in Fear Memory

**DOI:** 10.3390/antiox11050999

**Published:** 2022-05-19

**Authors:** Jiaxin Situ, Xuelian Huang, Mingyang Zuo, Yingying Huang, Bingyu Ren, Qiong Liu

**Affiliations:** 1 Shenzhen Key Laboratory of Marine Biotechnology and Ecology, College of Life Sciences and Oceanography, Shenzhen University, Shenzhen 518000, China; 2060251007@email.szu.edu.cn (J.S.); 2100251006@email.szu.edu.cn (X.H.); 2019301002@email.szu.edu.cn (M.Z.); 2020302013@email.szu.edu.cn (Y.H.); 2 Shenzhen-Hong Kong Institute of Brain Science-Shenzhen Fundamental Research Institutions, Shenzhen 518000, China; 3 Shenzhen Bay Laboratory, Shenzhen 518000, China; 4 Shenzhen Key Laboratory of Microbial Genetic Engineering, College of Life Sciences and Oceanography, Shenzhen University, Shenzhen 518000, China

**Keywords:** selenoprotein W, contextual fear conditioning, tandem mass tag (TMT)-based quantitative proteomics, oligodendrogenesis

## Abstract

The essential trace element selenium plays an important role in maintaining brain function. Selenoprotein W (SELENOW), the smallest selenoprotein that has been identified in mammals, is sensitive to selenium levels and abundantly expressed in the brain. However, its biological role in the brain remains to be clarified. Here, we studied the morphological and functional changes in the brain caused by SELENOW deficiency using its gene knockout (KO) mouse models. Histomorphological alterations of the amygdala and hippocampus, specifically in the female SELENOW KO mice, were observed, ultimately resulting in less anxiety-like behavior and impaired contextual fear memory. Fear conditioning (FC) provokes rapidly intricate responses involving neuroplasticity and oligodendrogenesis. During this process, the females generally show stronger contextual FC than males. To characterize the effect of SELENOW deletion on FC, specifically in the female mice, a Tandem mass tag (TMT)-based comparative proteomic approach was applied. Notably, compared to the wildtype (WT) no shock (NS) mice, the female SELENOW KO NS mice shared lots of common differentially expressed proteins (DEPs) with the WT FC mice in the hippocampus, enriched in the biological process of ensheathment and oligodendrocyte differentiation. Immunostaining and Western blotting analyses further confirmed the proteomic results. Our work may provide a holistic perspective of gender-specific SELENOW function in the brain and highlighted its role in oligodendrogenesis during fear memory.

## 1. Introduction

Fear, a complex cascade signaling in the autonomic nervous system triggered by threatening stimuli, is crucial to survival. Consolidation of fear memory requires the modulation of myelin plasticity and oligodendrogenesis [1] with hippocampal–amygdala circuits as the structural basis [2,3]. Gender differences have been reported during this process. In behavioral tests, such as contextual fear conditioning (CFC), more freezing time and stronger fear generalization in female mice than in male mice have been observed [4,5]. Dysregulation of fear memory may lead to chronic psychiatric disorders such as post-traumatic stress disorder (PTSD) and various types of phobias [6]. Recently, it has been reported that the PTSD-risk patients can benefit from selenium supplementation [7], but the mechanism remains to be clarified.

As an essential trace element with antioxidant effects, selenium plays a protective role in the brain’s response to stimuli [8]. The biofunction of selenium is achieved through selenocysteine, the 21st amino acid [9]. Proteins containing selenocysteines are selenoproteins. Selenoprotein W (SELENOW, former name SepW1) is known as the smallest one (~9.5 kDa) in the 25 currently identified selenoproteins [10,11], and it is highly regulated by selenium levels [12]. SELENOW has been found to express in various tissues with extremely high amounts in skeletal muscle, brain, heart, liver, and long bone [13,14]. Recently, a regulative role of SELENOW in osteoclastogenesis during bone remodeling has been discovered [14]. However, the function of SELENOW in the brain remains largely unknown, especially its involvement in fear memory regulation.

With a conserved selenocysteine-containing thioredoxin-like motif (CXXU) as its active sites, SELENOW is presumed to transduce hydrogen peroxide signals into regulatory disulfide bonds in specific target proteins [15]. In a former in vitro study, we identified a disulfide bond between the cysteine residues of SELENOW and microtubule-associated protein tau in the presence of hydrogen peroxide [16]. In addition, the reported subcellular location of SELENOW is partially in accordance with tau, which is in neuron axonal and dendritic processes [17,18]. In a study on the expression patterns of selenoproteins in mouse brain, SELENOW has been found to be particularly highly enriched in the cortical subplate (including amygdala), isocortex, and hippocampal formation [19], all of which are brain regions that are associated with fear memory. Furthermore, the expression of SELENOW is well maintained in the brain even in selenium deficiency cases [20], suggesting it may play an important role in the neurobiological process in those brain regions mentioned above.

As an effective tool for studying selenoproteins’ function, a conventional knockout (KO) mice model was used in our present study. Behavioral and histological studies on both of the male and female SELENOW KO mice were performed. We discovered that SELENOW deletion caused less anxiety-like behavior and impaired contextual fear memory, specifically in female mice at the age of 6 months. Histomorphological changes were found in fear-memory-related brain regions, including the amygdala and hippocampus, in female KO mice, to which we believed the behavioral changes may attribute. Then, tandem mass tag (TMT)-based quantitative proteomic analysis and validation were applied to better understand the molecular mechanism.

## 2. Materials and Methods

### 2.1. Animals

The homozygous SELENOW (Gene ID: 20364) knockout (referred as KO below) mice and wildtype (WT) mice (C57BL/6N) used as background control were generated and bred by BIOCYTOGEN (Beijing, China). The mice were housed and bred in cages under standard conditions with a 12:12 h light–dark cycle and a 22 °C room temperature.

### 2.2. Antibodies

Anti-SELENOW antibody was from Rockland Immunochemical (600-401-A29); anti-VGF, anti-MAP2, and anti-SYPL1 antibodies were from Abcam (Cambridge, UK; ab74140, ab5392, and ab184176); anti-IgMμ was from Sigma-Aldrich (Darmstadt, Germany; A8786); anti-GAPDH, anti-Beta Tubulin, and anti-MAP4 antibodies were from Proteintech (Rosemont, IL, USA; 11229-1-AP, 10068-1-AP, and 10494-1-AP); anti-MBP antibody was from Cell Signaling Technology (Danvers, MA, USA; 78896S). Peroxidase-conjugated anti-mouse/rabbit antibodies were from Abmart (Shanghai, China; m21001 and m21002), and peroxidase-conjugated anti-chicken and Alexa Fluor^®^ 488 anti-rabbit antibodies were from Jackson ImmunoResearch (West Grove, PA, USA; AB_2337381 and AB_2338046).

### 2.3. PCR Amplification

DNA was extracted from mouse tail using a nucleic acid purification kit (MF280-01, Mei5bio, Beijing, China), following the manufacturer’s protocol. The forward and reverse primers were 5’-CAGCTGGCTTGCAGTAAGTGATTG-3’ and 5’-GCCCCTGTTGCTGTTAGATTTCTTCA-3’, respectively. PCR was carried out as follows: initial denaturation at 95 °C for 3 min, the thermocycling profile at 32 cycles of denaturation at 95 °C for 15 s, annealing at 62 °C for 20 s, and extension at 72 °C for 50 s. Followed by a final extension of 7 min at 72 °C. The PCR products were examined on 1% agarose gel stained with ethidium bromide.

### 2.4. Brain Tissue Staining and Imaging

The 6 month old WT and KO mice were sacrificed, and their fresh separated brains were fixed in 4% polyformaldehyde (Sigma-Aldrich, Darmstadt, Germany) at 4 °C overnight. Then, their brains’ left hemispheres were dehydrated, embedded in paraffin wax or optimal cutting temperature (O.C.T) compound, and cut into 10 μm slides. The Nissl and Golgi staining were performed using Paraffin-embedded slides by Servicebio company (Wuhan, China). Immunostaining was performed using O.C.T.-embedded slides. They were blocked with 4% BSA at RT for 30 min, rinsed three times with PBS, then incubated overnight at 4 °C with the primary antibodies properly diluted in PBS containing 1% Triton X-100. After, the slides were incubated with Alexa-Fluor 488 secondary antibody at RT for 1 h, and counterstained with DAPI. After staining, the tissue slides were mounted with mounting medium and imaged under an OLYMPUS BX53 (Tokyo, Japan) microscope.

The spine numbers and length of the dendrites in hippocampus and amygdala neurons, and the MAP2 fluorescence intensity was measured by ImageJ software (version 1.53c). Spine numbers and dendritic length were measured using the Dendrite Spine Counter plugin. Generally, scales were firstly set for each image so that pixel information were converted to physical unit information. Then, dendritic segments were traced using the Polyline Tool and spines were automatically marked using Multi-Point Tool. The length of the dendritic segment (μm) and the number of spines along the segment were measured. Spine density was calculated as the spine numbers divided by dendritic length and then multiplied against 10 (numbers per 10 μm). For MAP2 fluorescence intensity in the hippocampus, each image was firstly converted to an 8 bit image format. Thresholds were set for DG (21, 255) and CA1 (24, 255), with the background set as dark. The area of MAP2 (as %) was measured for statistical analysis.

### 2.5. Blood Biochemical and Malondialdehyde (MDA) Analysis

Whole blood of each mouse was collected in 1.5 mL sterile tubes after sacrifice and processed as quickly as feasible. The samples were placed for 1 h at room temperature for a clot to form, and the supernatants were collected as serums for biochemical analysis. Biochemical indicators, including alanine transaminase (ALT), amylase (AMY), apolipoprotein B (ApoB), c-reactive protein (CRP), aspartate aminotransferase (AST), triglycerides (TG), uric acid (UA), and urea, were detected by an automatic biochemical analyzer (ICUBIO iMagic-M7, Shenzhen, China) with the corresponding kits.

The lipid peroxidation marker, MDA, in the cortex of 6 month old female WT and KO mice, was measured according to the instructions from commercially available kits (Solarbio, Beijing, China; BC0025). The cortex brain samples were weighed and lysed in extracting buffer. Homogenates were centrifugated, and then the supernatants were collected for MDA assays. Generally, the assays consisted of a two-stage process: enzymatic reaction, followed by chromogenic reaction. The absorbance was measured at the proper wavelength using a microplate reader (SYNERGY H1, BioTek Instruments Inc., Winooski, VT, USA).

### 2.6. Behavioral Tests

Six behavioral tests, including the open field test (OFT), elevated plus maze (EPM), novel object recognition (NOR), contextual fear conditioning (CFC), Y-maze, and Morris water maze (MWM), were applied to evaluate the anxiety degree, locomotor activity, learning ability, and memory capacity of the WT and KO mice.

Generally, the OFT tasks were performed in an L100 × W100 × H30 cm open-top chamber in a quiet room. The bottom of the chamber was divided into 25 squares with the size of L20 × W20 cm. The mice were placed in the center of the chamber and allowed to explore the open field for 5 min. For each mouse, the numbers of times of crossing grids, rearing, and defecation were recorded [21].

The EPM tasks were carried out in reference to a previous report with slight modifications [22]. The elevated plus maze apparatus was a cross-shaped maze that consisted of four arms with an L30 × W5 × H60 cm. Two closed arms were equipped with a 30 cm high wall, while two open arms were not. The mouse was initially placed facing an open arm in the center of the maze and then allowed to explore the maze for 5 min. The mice fell off the maze were excluded. The time of each mouse spent in the open and closed arms was recorded.

The NOR tasks were conducted in an L40 × W40 × H40 cm open-top square chamber in the test room equipped with a daylight lamp. This box was divided into 25 equal squares. In the habituation phase, each mouse was placed in the empty box for 5 min in order to habituate it to the environment and to the apparatus. The test sessions were performed the next day. In the acclimation phase, home cages were left for acclimation in the test room for 1 h prior to the beginning of the test. In the acquisition phase, each mouse was placed for 5 min in the box containing two identical objects. After a 90 min retention interval back to its home cage, the mouse was then placed into the box and exposed to one of the familiar objects and to a novel object for a short-term recognition memory test. The mouse was initially placed in the apparatus facing the wall and allowed to explore the objects for 5 min. When the mouse’s nose pointed or touched an object within 1 cm, it was recorded as an exploration behavior. The number of times the mouse explored one of the two objects was recorded. The discrimination ratio was defined as (T_new_ − T_old_)/T_total_; T_new_: exploration times for the new object, T_old_: exploration times for the old object, and T_total_: total exploration times.

The CFCs were conducted in two enclosed chambers (Sansbio, Jiangsu, China) equipped with top-view infrared cameras, decibel meters, controllable loudspeakers, white lights, and electric barriers. The mice freezing behavior was analyzed using tracking software (Sansbio, Jiangsu, China). On the first day (training stage), mice were placed in the chamber and recorded for 2 min as a baseline, and then they were trained with four pairs of tone (CS) and an electric foot shock (US). One pair of CS-US consisted of 30 s of tone (6000 Hz, 80 dB) followed by 2 s electric foot shock (0.35 mA). On the next day (testing stage), to test their contextual fear memory retention, the mice were re-exposed to the same conditioned chamber for 5 min without giving any tone and foot shock. One hour later, the mice were tested for novel condition fear memory and tune fear memory. The mice were exposed to an altered context with a different chamber shape and olfactory cues for 3 min as the novel condition fear memory. Then, the mice were given 30 s of tone (6000 Hz, 80 dB) as auditory cues, another 3 min were recorded as the tune fear memory. The percent freezing was defined as freezing time/total time [23]. Mice placed in the chamber without any electric foot shock were referred as no shock (NS), which were used as the negative controls for mice undergoing fear conditioning (FC) in the proteomic experiments.

The Y-maze tests were performed in a Y-shaped maze with three identical arms at a 120° angle from each other. The mice were placed in the center of the maze and were given free access to all three arms during a session lasting 5 min. If the mice chose a new arm over the one it visited previously, it was defined as an alteration. The total number of arm entries and the sequence of entries were manually recorded; then, the spontaneous alternation ratio and the number of total entries were analyzed [24]. The spontaneous alternation ratio was defined as N_alt_/(N_total_ − 2); N_alt_: number of alternations, an alternation was achieved when an animal entered a new arm rather than returning to one visited previously; N_total_: number of total arm entries.

The MWM tasks were performed in a water-filled round pool with the size of D120 × H50 cm [25]. The temperature of the water was kept at 22 ± 1 °C, and the depth of water was 26 cm. A camera was mounted on the top of the pool to track the moving trace of the mice. The pool was divided into four quadrants. A round plastic escape platform with a diameter of 12 cm was placed in one of the quadrants, with its surface 1–2 cm below the water level. Each mouse was manually guided to the platform for 15 s at first, and then placed in the opposite quadrant. The amount of time the mouse took to seek the platform was recorded by the tracking system within 60 s as the escape latency. The training trials were performed for five consecutive days. Then, after 24 and 72 h, the platform was removed. In addition, 60 s probe trials were conducted for assessing short- and long-term memory, respectively. The amount of time the mouse spent in the target quadrant and the number of times the mouse crossed the platform were recorded and analyzed by the SMART v3.0 (Panlab, Barcelona, Spain) software.

### 2.7. Tandem Mass Tag (TMT)-Based Quantitative Proteomics

The less anxious and impaired fear memory phenotypes of the SELENOW deletion was gender specific and only observed in female mice. Thus, quantitative proteomic analysis was applied to four groups of 6 month old female mice, including wildtype no shock (WT NS), wildtype fear conditioning (WT FC), SELENOW KO no shock (KO NS), and SELENOW KO fear conditioning (KO FC), to further explore the possible mechanisms. After fear conditioning, the female mice were sacrificed and their brain tissues, including hippocampus and amygdala, were rapidly separated on an ice-cold plate. The tissue samples were lysed, and the total protein concentrations were determined and optimized by a BCA protein assay kit (Thermo Scientific, Waltham, MA, USA). The TMT-based quantitative proteomics were completed by Wininnovate Bio (Shenzhen, China). Briefly, 200 μg protein lysates from each sample were subjected to proteolytic digestion by trypsin (Promega, Madison, WI, USA) using a filter-aided sample preparation (FASP) method as described by Wiśniewski, Jacek R. et al. [26].

The digested peptides were labeled with reagents from a TMT-10plex kit (Thermo Scientific, Waltham, MA, USA). The digested peptides were eluted and combined into 20 fractions by high pH reverse-phase (RP) chromatography using the Easy nLC 1200 system (Thermo). Then, they were lyophilized for LC-MS/MS. Data-dependent acquisition (DDA) mass spectrum techniques were used to acquire tandem MS data on a ThermoFisher Q Exactive mass spectrometer (Thermo Scientific, Waltham, MA, USA) fitted with a Nano Flex ion source. Data were acquired using an ion spray voltage of 1.9 kV and an interface heater temperature of 275 °C. The MS was operated with FULL-MS scans. For the DDA, survey scans were acquired in 250 ms and up to 20 product ion scans (50 ms) were collected. Only spectra with a charge state of 2–4 were selected for fragmentation by higher-energy collision energy. Dynamic exclusion was set for 25.

### 2.8. Western Blotting

Whole cell or tissue extracts from the 6 month old female WT and KO mice were prepared by lysing in commercial WB and IP lysis buffer from Beyotime (P0013, with protease inhibitors cocktail) on ice for 30 min and then ultrasonication. The lysates were spun at 20,000× *g* for 15 min at 4 °C. The supernatants were then used in Western blotting experiments. BCA protein assay kits (Thermo Scientific, Waltham, MA, USA) were used to measure the protein concentration, and equal amounts of 30 μg total proteins were loaded onto SDS-PAGE gel. Proteins were transferred to a PVDF membrane at 100 V for 90 min. Blots were blocked in TBST with 5% nonfat dried milk for 2 h and incubated with primary antibodies at appropriate concentration at 4 °C overnight. Blots were then rinsed 3 × 15 min in TBST and incubated with peroxidase-conjugated secondary antibody (1:5000) for 2 h at room temperature. Membranes were incubated with Advansta ECL (Menlo Park, CA, USA; K-12045-D50) and imaged under a Tanon (Shanghai, China) 5200 imager for chemiluminescence detection. The band intensity was quantified by ImageJ (version 1.53c). The obtained images were firstly converted to 8 bit format to perform uncalibrated optical density (OD). The background was subtracted through the rolling ball radius method. Each blotting band was individually selected and circumscribed with the rectangular ROI selection and “Gels” function, followed by quantification of peak area of obtained histograms. Data were acquired as an arbitrary area values for statistical analysis.

### 2.9. Statistical Analysis

For proteomics data, the MS/MS data were analyzed for protein identification and quantification using Proteome discoverer (v2.5). The local false discovery rate was 1.0% after searching against Homo sapiens protein database with a maximum of two missed cleavages and one missed termini cleavage. The following settings were selected: oxidation (M), acetylation (protein N-term), deamidation (NQ), pyro-glu from E, pyro-glu from Q for variable modifications as well as carbamidomethylation (C), TMT-10plex (N-term), and TMT-10plex (K) for fixed modifications. Precursor and fragment mass tolerance were set to 10 ppm and 0.05 Da, respectively. Proteins with abundances that changed <1.2-fold and *p* > 0.05 were discarded. Proteins with Log2 ratios of either ≥0.263 or ≤ −0.263 with *p* < 0.05 were identified as differentially expressed proteins (DEPs).

For other assays, all of the data are representative of at least three independent experiments. Data are presented as the mean ± SEM. Statistical comparisons were performed using a Student’s *t*-test, one-way ANOVA followed by Bonferroni’s multiple comparison test, or two-way ANOVA followed by Sidak’s multiple comparisons test. * *p* < 0.05, ** *p* < 0.01, and *** *p* < 0.001 were considered to be statistically significant.

## 3. Results

### 3.1. Less Anxiety-like Behavior and Impaired Contextual Fear Memory in SELENOW KO Female Mice

**Figure 1:**
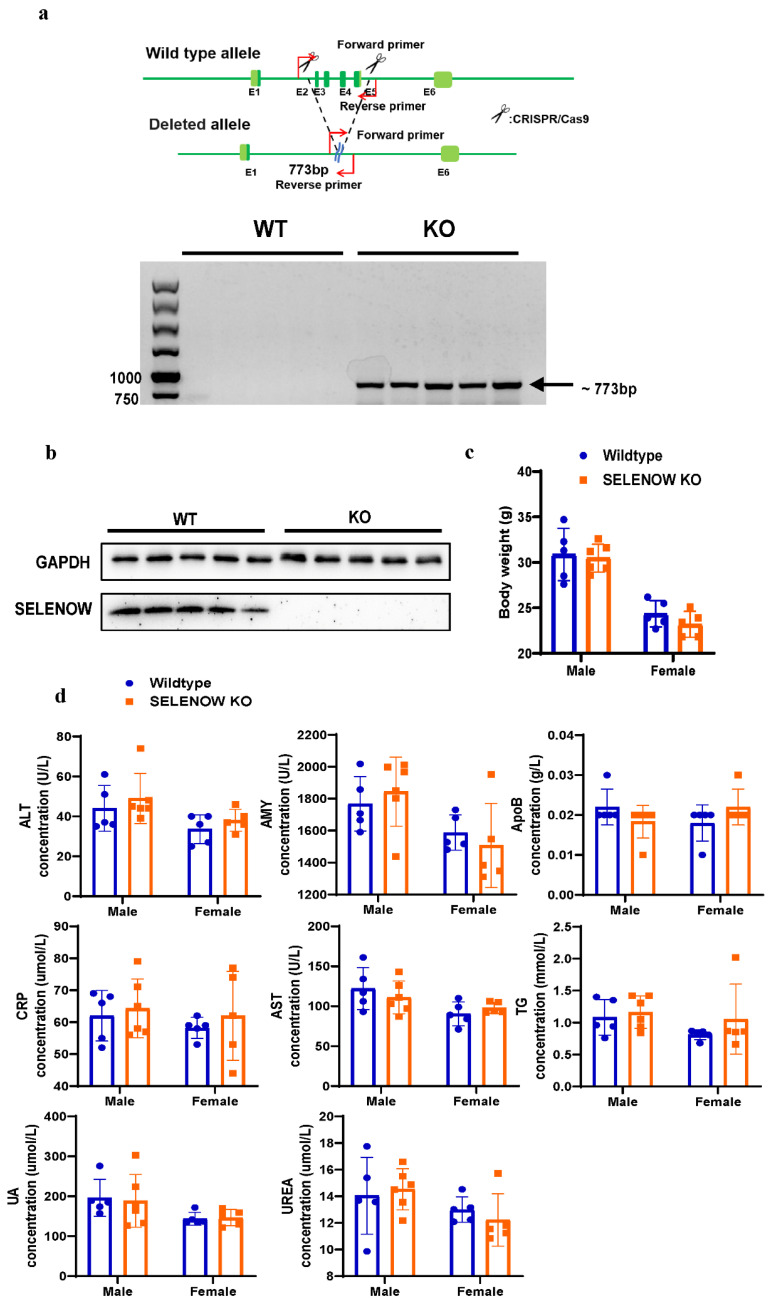
Generation and characterization of SELENOW knockout mice. (**a**) Representative PCR amplification results of the tails of 6 month old wildtype (WT) and SELENOW knockout (KO) mice (3 males and 2 females). The primers were designed to amplify positive bands (approximately 773 bp) in KO templates as shown in the schematic diagram. (**b**) Representative Western blotting of SELENOW expression in the cortex brain tissue of 6 month old wildtype (WT) and SELENOW knockout (KO) mice (3 males and 2 females). The GAPDH was used as the housekeeping protein. (**c**) Body weight of 6 month old WT and SELENOW KO mice (*n* = 5 or 6). No significant statistical difference was found between WT and KO mice in the same gender. (**d**) Blood biochemical parameters of 6 month old WT and SELENOW KO mice (*n* = 5 or 6). No significant statistical difference was found between WT and KO mice in the same gender. ALT: alanine transaminase; AMY: amylase; ApoB: apolipoprotein B; CRP: c-reactive protein; AST: aspartate aminotransferase; TG: triglycerides; UA: uric acid. Data are expressed as the mean ± SEM (two-way ANOVA followed by Sidak’s multiple comparisons test).

The abundance of SELENOW in the brain indicated it may play a role in the nervous system. To study the potential involvement of SELENOW in brain function, the SELENOW knockout (KO) mice were generated. Specific primers targeting the flanking sequences of exon 2 to exon 5 of the SELENOW gene were designed to identify the KO mice as shown in the schematic diagram in Figure 1a. The PCR of homozygous KO resulted in 773 bp shift-mutated fragments (Figure 1a). Then, the immunoblotting results, shown in Figure 1b, detected the absence of SELENOW protein in the brain tissue of the 6 month old KO mice compared with wildtype (WT), further confirming that SELENOW was successfully knocked out. The homozygous KO mice were fertile and exhibited no obvious difference in size and body weight compared with the age- and sex-matched WT (Figure 1c). Biochemical parameters evaluating the health and metabolism state of the whole body were detected using serums from KO and WT mice, and no significant difference was found (Figure 1d).

**Figure 2:**
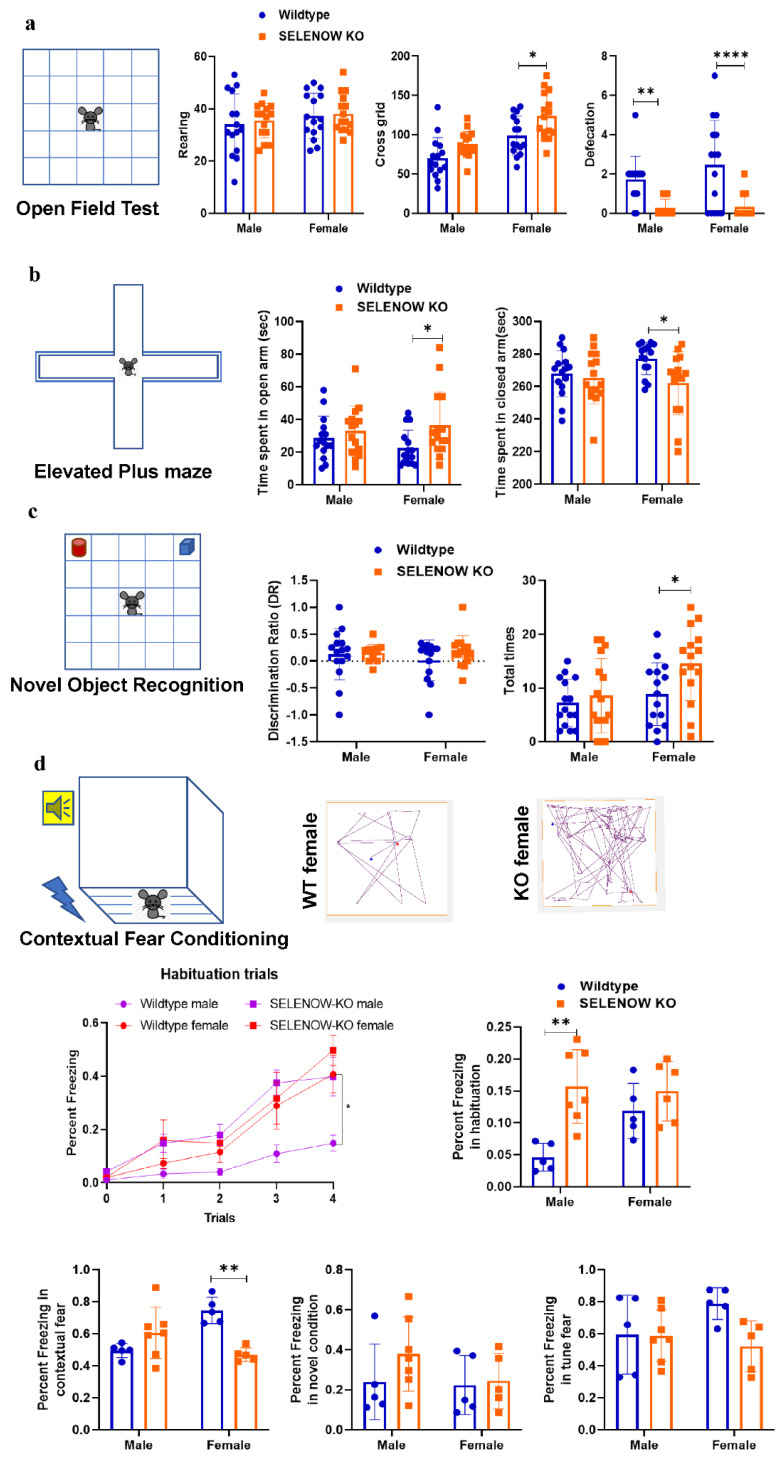
Behavioral test results for the assessment of locomotion ability, anxiety, and learning and memory in WT and KO mice. (**a**) Locomotion ability and anxiety of the 6 month old WT and KO mice evaluated by the crossing grid numbers, rearing and defecation times in the open field test (*n* = 15 mice). (**b**) Anxiety in the 6 month old WT and KO mice was evaluated by the time spent in the open arms and closed arms of the elevated plus maze test (*n* = 15 mice). (**c**) Declarative memory of the 6 month old WT and KO mice evaluated by the discrimination ratio and total times in the novel object recognition task (*n* = 15 mice). (**d**) Fear memory of the 6 month old WT and KO mice evaluated by the percent freezing in habituation, contextual fear, novel condition, and tune fear stages in the contextual fear memory task. The representative track trails of female WT and KO mice in the contextual fear stage were also provided (*n* = 5 or 7 mice). Data are expressed as the mean ± SEM (two-way ANOVA followed by Sidak’s multiple comparisons test). * *p* < 0.05; ** *p* < 0.01, **** *p* < 0.0001.

To identify the function of SELENOW in the brain, the 6 month old KO and WT mice underwent several behavioral tests including OFT, EPM, NOR, CFC, Y-maze, and MWM. As shown in Figure 2a, the female KO mice demonstrated a significant increase in the number of grid crossings in the OFT compared with female WT (*p* = 0.0149), and the defecation times of both male (*p* = 0.0076) and female (*p* < 0.0001) KO mice were obviously less than their matched WT. No change in the rearing frequencies was detected (male: *p* = 0.8666; female: *p* = 0.9616). In the EPM and NOR tests, the female KO mice exhibited an increased tendency to explore the open arms (*p* = 0.0340) and objects (*p* = 0.0260) compared with female WT mice. Meanwhile, no difference in the discrimination ratio was detected between the WT and KO (male: *p* = 0.9812; female: *p* = 0.3848). The Y-maze and MWM tests obtained no significant difference between the WT and KO in the spontaneous alternation ratio, number of total arm entries, escape latency, time spending in the target quadrant (24/72 h), the number of times the mice crossed the platform (24/72 h), mean speed, and total distance. The results are provided in Supplementary Figures S1 and S2. In the CFC training stage, both WT and KO mice showed an increased freezing percentage during the habituation (male: *p* = 0.0012; female: *p* = 0.4867), revealing normal learning ability in the training session (Figure 2d). Yet, there was a significant difference in freezing percentages between male WT and KO mice at trial 4 (*p* = 0.0225), indicating a lower fear response in the male WT. In the CFC testing stage, no significant difference was found in the novel condition and tune fear memory tests, while an obvious decrease in the freezing percentage in the contextual fear memory test was detected in the female KO mice compared with female WT (*p* = 0.0010). Altogether, the above behavioral tests indicated that the anxiety degree of the 6 month old female KO mice decreased, and the fear memory was impaired. Meanwhile, their spatial working and learning memories seemed to be unaffected.

### 3.2. Histomorphologic Alteration of Amygdala and Hippocampus in SELENOW KO Female Mice

**Figure 3:**
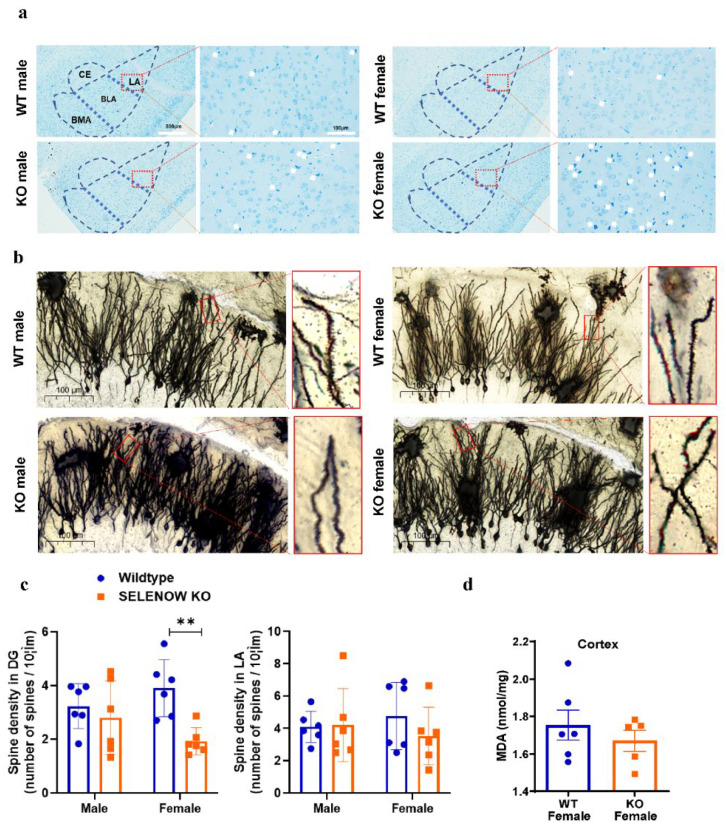
Staining and MDA detection of representative brain sections in WT and KO mice. (**a**) Nissl staining of amygdala in 6 month old WT and KO mice. The subregions of the amygdala, including the lateral amygdala (LA), basolateral amygdala (BLA), basomedial amygdala (BMA), and central amygdala (CE), are shown in the blue, dashed lines. Images on the right are LA areas, enlarged in the red box. White arrows indicate the small and darkly stained injured neurons that were characterized by cytoplasmic shrinkage, nuclear pyknosis, and hyperchromasia. (**b**) Golgi staining of the hippocampus DG area and typical dendrites in 6 month old WT and KO mice. (**c**) Statistics and analysis of spine density in DG and LA. The dendrite length and spine numbers were measured by ImageJ. Spine density was defined as the spine numbers per 10 μm of dendritic length (*n* = 6). Data are expressed as the mean ± SEM (two-way ANOVA followed by Sidak’s multiple comparisons test). ** *p* < 0.01. (**d**) MDA levels in the cortex of 6 month old female WT and KO mice. Data are expressed as the mean ± SEM (Student’s *t*-test, *n* = 6 in WT and *n* = 5 in KO).

Since the hippocampal–amygdala circuit has been identified as the major brain pathway that is responsible for retrieval of contextual fear memory [27], we performed Nissl and Golgi staining to characterizing the possible morphological changes of these two brain regions in KO. In the amygdala of female KO mice, abnormal Nissl bodies and obvious neuronal damage were detected as shown in Figure 3a (white arrows). The dendrite spine density of the functional neurons in this region remained unaffected (Figure 3c). Unlike the amygdala, no morphological changes were found in the hippocampus by Nissl staining (Supplementary Figure S3), and a significant decrease in the dendritic spine density (*p* = 0.0095) in the dentate gyrus (DG) of the female KO mice was discovered (Figure 3b,c). Since the protective role of SELENOW in oxidative stress has been emphasized in the reports of several knockdown models [28,29], levels of MDA, a lipid peroxidation marker, were also detected in the cortex of female WT and KO mice. As shown in Figure 3d, no significant difference was found (*p* = 0.4315).

### 3.3. Identification of DEPs by TMT-Based Quantitative Proteomic Analysis

**Figure 4:**
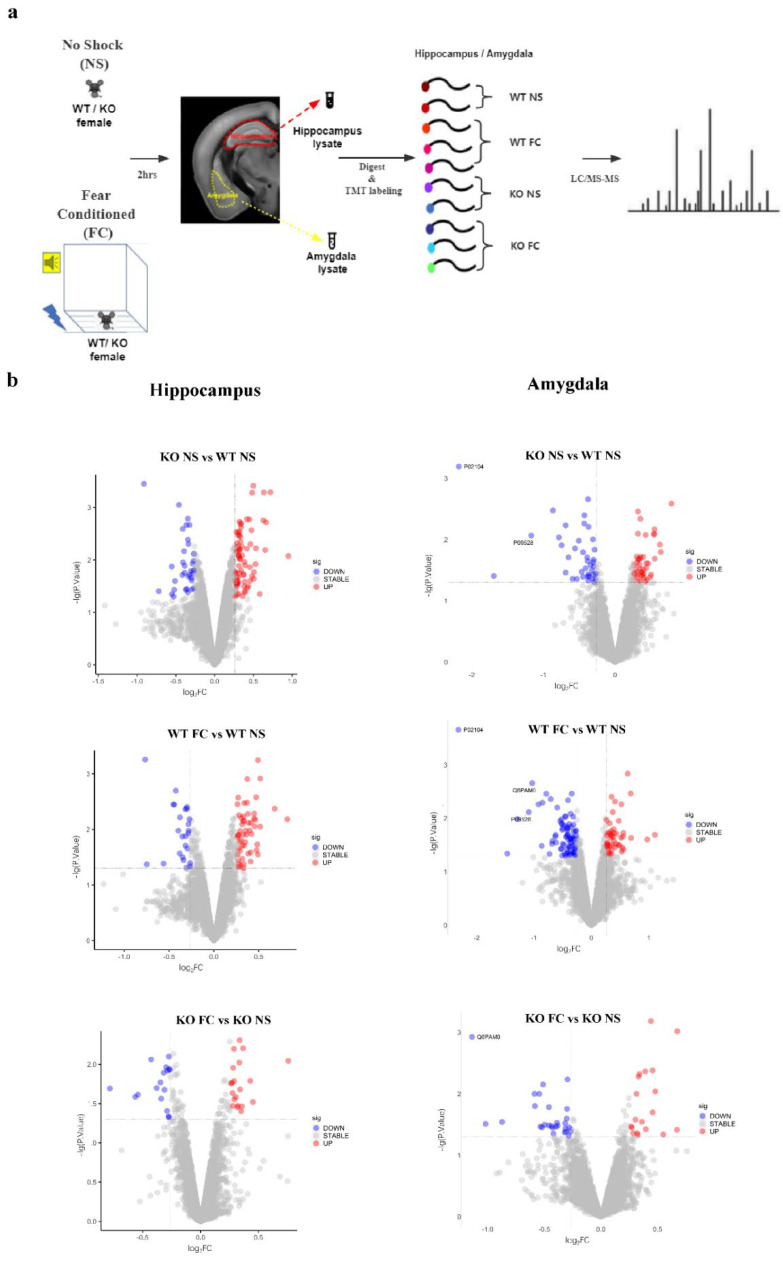
The overall design and differentially expressed proteins (DEPs) detected by TMT-based proteomics. (**a**) The schematic diagram of fear conditioning, protein collection, and TMT labeling. The hippocampus or amygdala of the right hemispheres from 4 female mice were collected for labeling in the WT NS and KO NS group, respectively (*n* = 4, the tissue from 2 mice were mixed and labeled with one TMT reagent). Hippocampus or amygdala of the right hemispheres from 6 female mice were collected for WT FC and KO FC group, respectively (*n* = 6, the tissue from 2 mice were mixed and labeled with one TMT reagent). NS: no shock, FC: fear conditioning, WT: wildtype, KO: SELENOW knockout. (**b**) The volcano plots (−log10 *p*-value vs. log2 fold change) of identified DEPs in the hippocampus (left panel) and amygdala (right panel). Proteins with log2 fold change either ≥0.263 or ≤ −0.263 and *p*-value < 0.05 were identified as DEPs (the upregulated DEPs were displayed as red dots, and the downregulated DEPs were displayed as blue dots).

As described above, only the female KO mice displayed the phenotype of fear memory deficit with histomorphological alteration in amygdala and hippocampus. To further elucidate which proteins and pathways were affected by SELENOW deletion in the contextual memory retrieval process of female mice, we conducted TMT-based proteomic analysis for both the hippocampus and amygdala collected after the CFC testing stage. The group design and the workflow of the TMT proteomic experiments are presented in Figure 4a.

For hippocampal proteomics, a total of 42,404 peptide fragments were identified and 5792 quantifiable proteins were obtained. For amygdala proteomics, a total of 38,985 peptide fragments were identified and 5309 quantifiable proteins were obtained. In order to analyze DEPs between two groups, data were further screened by differences in fold changes. A protein was identified as DEP if its fold change (FC) was >1.2 (down < 0.83 times or up > 1.2 times), and the *p*-value was <0.05 relative to the control group. The volcano plots are provided in Figure 4b.

Based on the above criteria, in the hippocampus, a total of 112 proteins were found to be differentially expressed as a result of SELENOW deletion in female mice (KO NS vs. WT NS), including 77 upregulated DEPs and 35 downregulated DEPs. Next, 79 DEPs (54 up-regulated and 25 down-regulated) were detected between WT NS and WT FC, while 37 DEPs (20 upregulated and 17 downregulated) were identified between KO FC and KO NS. There were obviously fewer proteins that were differentially expressed in the KO hippocampus compared with WT after fear conditioning.

Similar cases were found in the amygdala results. A total of 83 proteins had been found to be differentially expressed as a result of SELENOW deletion in the female mice (KO NS vs. WT NS), including 43 upregulated DEPs and 40 downregulated DEPs. One hundred and thirty-four DEPs (48 upregulated and 86 downregulated) were detected between WT NS and WT FC, while 47 DEPs (19 upregulated and 28 downregulated) were identified between KO FC and KO NS. The detailed information, including the Uniprot ID, full name, fold change, and *p*-value, of the above DEPs were provided as lists in the Supplementary Tables S1–S6.

### 3.4. Analysis and Comparison of Protein Expression Profiles

**Figure 5:**
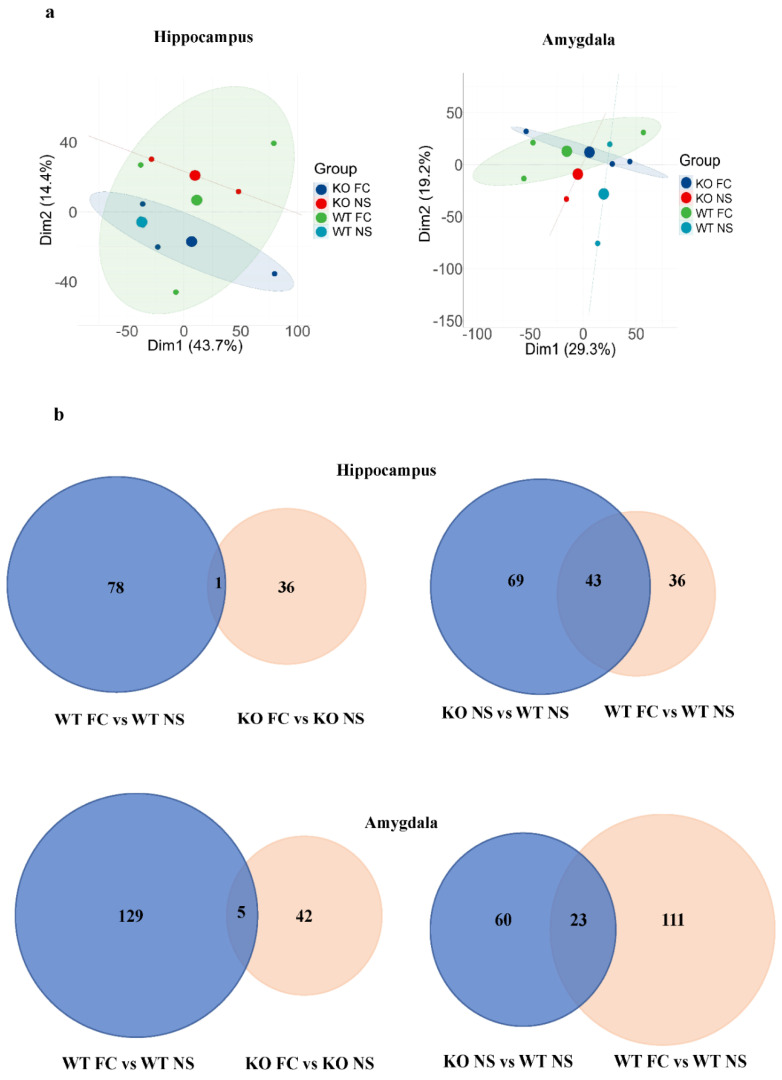
Female SELENOW KO NS and WT FC shared common features. (**a**) Principal component analysis (PCA) of the hippocampus (left panel) and amygdala (right panel) proteomic results. (**b**) The Venn diagram of the DEPs from WT FC vs. WT NS groups overlapping with the DEPs from the KO FC vs. KO NS groups (left), and DEPs from the KO NS vs. WT NS groups overlapping with DEPs from the WT FC vs. WT NS groups (right). Upper panel: hippocampus; lower panel: amygdala.

To gain a preliminary understanding of the proteomic data, principal component analysis (PCA) was applied. As shown in Figure 5a, the experimental and control samples could be discriminated, revealing the reliability of the data. Notably, the KO NS group and WT FC group and the KO FC group and WT NS group could be clustered together in the hippocampus results, suggesting the groups that were clustered together may share similar protein expression profiles in the hippocampus.

To further illustrate the similarities and differences among DEPs caused by SELENOW deletion and fear conditioning, Venn diagrams were presented (Figure 5b). In the hippocampus results, the number of proteins that were differentially expressed in WT was about two-fold more than the number in KO after fear conditioning. As listed in Table 1, they only shared one protein in common, synaptophysin-like protein 1 (O09117). Furthermore, its expression was upregulated in WT but downregulated in KO after fear conditioning.

In the amygdala results, the number of proteins that were differentially expressed in WT was approximately 2.8-fold more than the number in KO after fear conditioning. They shared five proteins in common, which were Ig mu chain C region (A0A075B6A0), choline transporter-like protein 2 (A0A1L1SU40), potassium voltage-gated channel subfamily B member 1 (Q03717), 5’-AMP-activated protein kinase subunit beta-2 (Q6PAM0), and THUMP domain-containing protein 1 (Q99J36). Their fold changes showed the same trend in both WT and KO after fear conditioning.

### 3.5. DEPs Common in Female SELENOW KO and Fear Conditioning WT Mice and Their Gene Ontology Categorization

**Figure 6:**
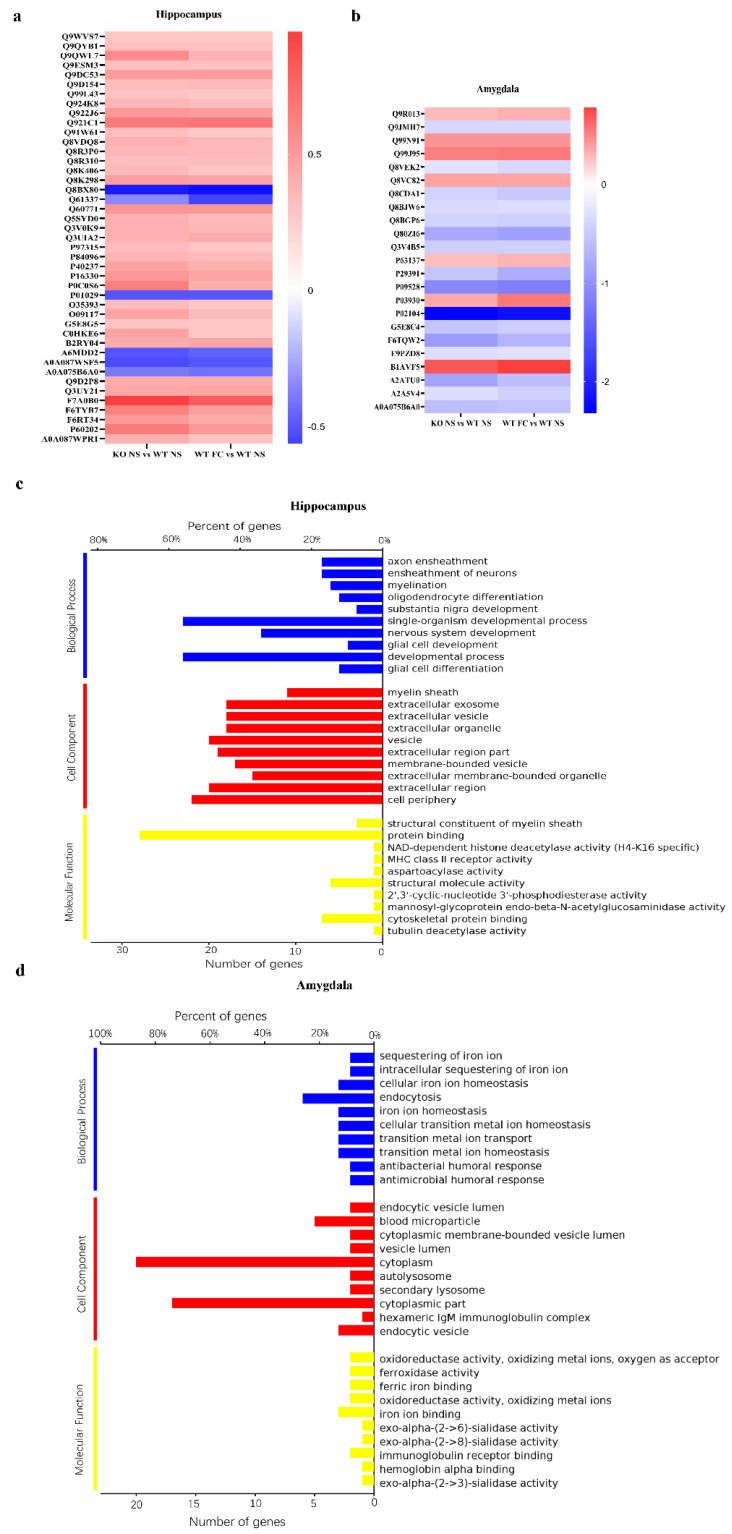
Common DEPs shared by the KO NS and WT FC mice and their GO analysis. Heatmaps of common DEPs shared by the KO NS and WT FC mice compared with the WT NS in the hippocampus (**a**) and amygdala (**b**). The Uniprot ID of these DEPs are provided on the left, and the rectangles with a gradient color from red to blue were for log2 fold change. The GO analysis of common DEPs shared by KO NS and WT FC compared with WT NS in the hippocampus (**c**) and amygdala (**d**).

Of note was that KO NS shared many common DEPs with WT FC when they were both compared with WT NS. In the hippocampus, there were 43 shared DEPs, and in the amygdala, the result was 23 (Figure 5b, right panel). Then, we studied the expression patterns of these shared DEPs. As displayed in the heatmaps in Figure 6a,b, all the common DEPs showed the same change trend, without exception. The detailed information, including Uniprot ID, full name, and fold change, of these shared DEPs were provided as listed in the Supplementary Tables S1–S6. It indicated that the effect of the SELENOW deletion may have something in common with the effect of fear conditioning.

To gain comprehensive information on the function, localization, and biological pathways of these shared DEPs, they were categorized through Gene Ontology (GO). GO annotated a gene product with respect to three aspects including biological process (BP), cell component (CC), and molecular function (MF) [30]. Figure 6c,d showed two GO analysis overview graphs of shared DEPs from the hippocampus and amygdala, respectively. The cut-off of the *p*-value was set to 0.05. Up to 10 significantly enriched terms in the BP, CC, and MF categories are displayed, and the terms of the same category were ordered by *p*-values.

For biological process categories, the common DEPs in the hippocampus were enriched in the axon ensheathment, ensheathment of neurons, myelination, oligodendrocyte differentiation, substantia nigra development, single-organism developmental process, nervous system development, glia cell development, developmental process, and glia cell differentiation. It is worth noting that most of these processes were fully or partially related with oligodendrogenesis. The shared DEPs in the amygdala were enriched in the sequestering of iron ion, intracellular sequestering of iron ion, cellular iron ion homeostasis, endocytosis, iron ion homeostasis, cellular transition metal ion homeostasis, transition metal ion transport, transition metal ion homeostasis, antibacterial humoral response, and antimicrobial humoral response.

In the category of cellular component, the top 10 significantly enriched terms in the hippocampus were myelin sheath, extracellular exosome, extracellular vesicle, extracellular organelle, vesicle, extracellular region part, membrane-bounded vesicle, extracellular membrane-bounded organelle, extracellular region, and cell periphery.The top 10 significantly enriched terms in the amygdala were endocytic vesicle lumen, blood microparticle, cytoplasmic membrane-bounded vesicle lumen, vesicle lumen, cytoplasm, autolysosome, secondary lysosome, cytoplasmic part, hexameric IgM immunoglobulin complex, and endocytic vesicle.

Regarding the molecular function, enrichment analysis showed that shared DEPs from the hippocampus were distributed in the structural constituent of the myelin sheath, protein-binding NAD-dependent histone deacetylase activity, MHC class II receptor activity, aspartoacylase activity, structural molecule activity, 2’,3’-cyclic-nucleotide 3’-phosphodiesterase activity, mannosyl-glycoprotein endo-beta-N-acetylglucosaminidase activity, cytoskeletal protein binding, and tubulin deacetylase activity. Shared DEPs from the amygdala were distributed in oxidoreductase activity, oxidizing metal ions, oxygen as acceptor, ferroxidase activity, ferric iron binding, oxidoreductase activity, oxidizing metal ions, iron ion binding, exo-alpha-(2->6)-sialidase activity, exo-alpha-(2->8)-sialidase activity, immunoglobulin receptor binding, hemoglobin alpha binding, and exo-alpha-(2->3)-sialidase activity.

### 3.6. Validation of Proteomic Results Revealing Abnormal Synaptic Plasticity and Oligodendrogenesis in Female SELENOW KO Brain

**Figure 7:**
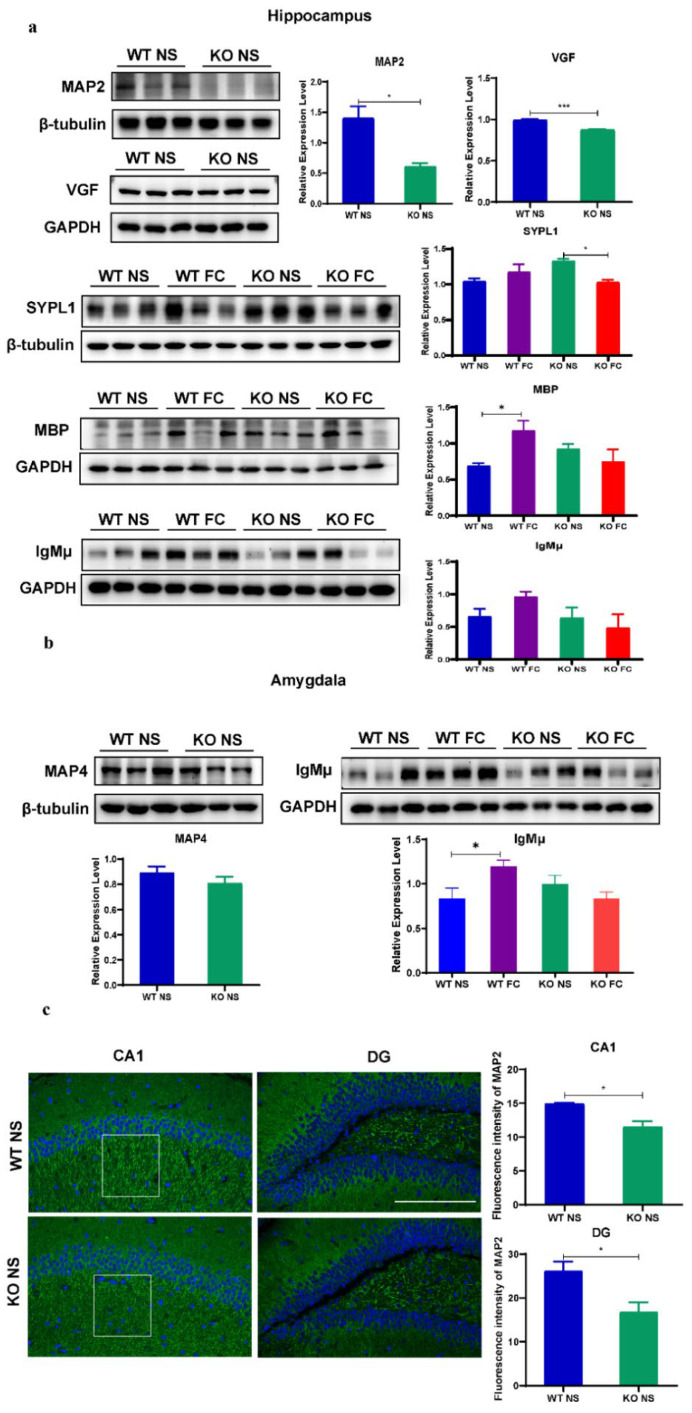
Validation of the proteomic data. (**a**) Representative Western blots and quantified data of the selected DEPs including MAP2, VGF, SYPL1, MBP, and IgMμ from the hippocampus data. Data are presented as the means ± SEM (one-way ANOVA followed by Bonferroni’s multiple comparison test or Student’s *t*-test, *n* = 3). * *p* < 0.05 and *** *p* < 0.001. (**b**) Representative Western blots and quantified data of selected DEPs including MAP4 and IgMμ from the amygdala data. Data are presented as the means ± SEM (one-way ANOVA followed by Bonferroni’s multiple comparison test or Student’s *t*-test, *n* = 3). * *p* < 0.05. (**c**) Representative immunostaining images of MAP2 (bright green) and nucleus (stained with DAPI in blue) in the hippocampus of WT NS and KO NS brain slides, and quantitative analysis of MAP2 fluorescence intensity in CA1 and DG areas. * *p* < 0.05. Scale bar = 200 μm. Data are expressed as the mean ± SEM (*n* = 3, Student’s *t*-test).

To validate the proteomic data, immunoblotting and immunostaining approaches were applied. Together, we selected five proteins in the hippocampus results. As shown in Figure 7a, MAP2 and VGF were proteins that were significantly downregulated caused by the SELENOW deletion (KO NS vs. WT NS). MBP, SYPL1, and IgM (μ chain) were proteins that were upregulated both after the SELENOW deletion and fear conditioning. In addition, two proteins from the amygdala results were selected for validation (Figure 7b). MAP4 was downregulated by the SELENOW deletion, and IgM (μ chain) was upregulated both by the SELENOW deletion and fear conditioning. The change trends in the protein expression level of these proteins were consistent with proteomic results. In Figure 7a, *p* = 0.0151 for MAP2, *p* = 0.0003 for VGF, *p* = 0.0458 for SYPL1 KO NS vs. KO FC, *p* = 0.0354 for MBP WT NS vs. WT FC. In Figure 7b, *p* = 0.0379 for IgMμ WT NS vs. WT FC.

We also performed immunostaining to detect the distribution and expression of MAP2 in the hippocampus of WT NS and KO NS. In accordance with the immunoblotting results, MAP2 fluorescence signals in the hippocampus cornu ammonis 1 (CA1) and dentate gyrus (DG) subregions of the KO NS were obviously less than for the WT NS (*p* = 0.0142 in CA1 and *p* = 0.0360 in DG). Meanwhile, the CA1 subregion displayed disruption of axonal and dendritic cytoarchitecture with fragmented, scattered, and unaligned signals in KO NS (Figure 7c, white boxes).

## 4. Discussion

Recently, two functional studies that were also based on the genetic knockout SELENOW mice models have been reported [14,31]. However, their observations mainly focus on the male mice, and the phenotypes in relation with brain function were not reported. In this study, we discovered gender-specific SELENOW deletion phenotypes, which were brain anatomical alterations with impaired fear memory only shown in female mice. Usually, the female mice showed more fear response to the same context and stronger contextual fear conditioning than the male mice [4,5]. Though a study in rodents detected no differences in brain SELENOW protein levels between male and female [32], our results indicated that the role of SELENOW in the brain is different between genders. Female mice with lower SELENOW protein levels may have a higher risk of fear memory impairment. A similar gender difference has also been well recognized in disease with memory impairment such as Alzheimer’s disease (AD) [33]. The act of follicle-stimulating hormone (FSH) was found to be one of the possible reasons for the higher incidence of AD in female [34], whether similar mechanism lying behind the fear memory impairment of our female SELENOW KO mice remains to be studied in the future.

Unlike the phenotypes of other selenoproteins’ knockout, such as GPX, selenoprotein F, T, and P, which have been reported to be associated with glucose and lipid metabolism [35,36,37], no such alternation has been observed in our SELENOW KO mice so far. The KO mice had the same body size and weight as the WT mice, and blood biochemical analysis indicated normal lipid metabolism and function of liver, kidney, and pancreas in KO. Similarly, a study by Min-Gyeong Shin et al. reported no significant change in insulin sensitivity and H_2_O_2_ levels in the SELENOW KO mice skeletal muscle, and SELENOW deficiency did not aggravate the high-fat diet feeding-induced insulin resistance and oxidative stress [31].

The proteomic and Western blot analyses revealed that the neuronal marker MAP2 was significantly decreased in the hippocampus of female KO, and the immunostaining further presented a disruption in the dendritic cytoarchitecture in its CA1 subregion. This was in accordance with the reduction in the dendrite spine density that we observed by Golgi staining. Interestingly, the SYPL1, a protein that locates in the synaptic vesicle membrane and is related to the neuroendocrine-specific synaptophysin [38], was upregulated in the hippocampus of female KO. We speculated it may be a compensatory mechanism against the synaptic defects. Therefore, unlike female WT mice, SYPL1 expression was unable to be further upregulated in female KO mice after fear conditioning.

A noticeable point was that SELENOW seemed to be related with several members of the microtubule associated protein (MAP) family, which play integral roles in the microtubule organization, remodeling, and stabilization in brain development and function [39]. MAP2 was downregulated in the hippocampus of female KO, and in the amygdala proteomic results, MAP4 was identified as one of the downregulated DEPs. Compared with MAP2, MAP4 is more ubiquitously distributed in various tissues and lowly expressed in the developmental brain [40]. But it is subsequently upregulated in the adult brain, mainly enriched in the axons, and potentially modulates the property of microtubules and actin neurofilaments [41]. In addition, we previously discovered that SELENOW may negatively regulate the microtubule-associated protein tau (MAPT, tau) accumulation, and identified a disulfide bond mediated by the cysteine-37 of SELENOW and cysteine-322 of tau [16]. The cysteine-322 is located in highly conserved microtubule-binding repeats of the C-terminal domain, which tau, MAP2, and MAP4 all share [42]. The mechanism for SELENOW in tau homeostasis regulation, and whether it is universal for other microtubule-associated proteins like MAP2 and MAP4 remains to be investigated.

Promotion of oligodendrogenesis and de novo myelination have been found to be of great importance in experience-driven memories such as fear memory [43]. Indeed, biological process of myelination and oligodendrogenesis were observed in our female WT mice after fear conditioning. Several proteins that were specific to oligodendrocytes including the myelin basic protein, myelin proteolipid protein, myelin-associated glycoprotein, myelin-oligodendrocyte glycoprotein, and myelin-associated oligodendrocyte basic protein were found upregulated in the proteomic data, together with aspartoacylase, a key enzyme that supports myelination [44]. Of note, the naive female KO exhibited similar myelinating features like the fear conditioned WT. It has been proved that prevention of oligodendrogenesis may lead to fear memory impairment [45,46]; thus, we speculated that female KO failed to further activate the oligodendrogenesis process after fear conditioning, which may ultimately result in fear memory deficit.

Oxidative stress may disrupt oligodendrocyte myelination and maturation [47]. Compared with mature myelinating oligodendrocytes, immature oligodendrocytes showed higher susceptibility to oxidative stress and inflammation [48]. SELENOW has been proposed as a glutathione-dependent antioxidant [49] and exhibits an immediate response to oxidative stress [50]. In our study, knockout of SELENOW had no significant effect on the MDA levels in the cortex of female mice, which indicated that oxidative stress may not be a typical phenotype of female SELENOW KO. This was not unexpected, since the relationship between SELENOW and oxidative stress was more likely a one-sided one. Many studies found the upregulation of SELENOW during oxidative stress, but SELENOW depletion or deletion did not cause oxidative stress in turn. For example, a former study has also reported that though SELENOW is important for the antioxidative system, oxidative stress was not a causative factor for the apoptosis of SELENOW-depleted skeletal muscle cells [51]. Notably, a positive association had been found between oxidative stress and fear conditioning [52]. Thus, it is still worth exploring whether SELENOW is involved in the determination of oligodendrocytic fate by modulating cellular redox status during fear memory process.

## 5. Conclusions

In conclusion, this study is the first to report SELENOW’s brain function in fear memory regulation. We discovered that the 6 month old female SELENOW KO mice exhibited less anxiety-like behavior in the OFT, EPM, and NOR tests, together with impaired contextual fear memory in the CFC. Furthermore, SELENOW deletion provoked anatomical alterations including obvious neuronal damage in the amygdala and decreased dendritic spine density in the hippocampus. Proteomic analysis and validation data emphasized the downregulation of neuronal protein and upregulation of myelination-related proteins in the hippocampus of female SELENOW KO mice. Thus, the altered protein profile may negatively affect synaptic regulation and fail to further support the oligodendrogenesis process during fear conditioning, ultimately leading to fear memory impairment. Our results revealed the physiological role of SELENOW in memory formation and may help us to better understand the mechanism underlying the treatment of selenium in fear-dysregulated disorders like PTSD.

## Figures and Tables

**Table 1:** Proteins that were both regulated in WT and KO after fear conditioning.

Brain Region	Uniprot ID	Name	Fold Change	
			WT FC vs. WT NS	KO FC vs. KO NS
Hippocampus	O09117	Synaptophysin-like protein 1 (SYPL1)	1.26	0.83
Amygdala	A0A075B6A0	Ig mu chain C region (IgMμ)	0.66	0.69
	A0A1L1SU40	Choline transporter-like protein 2 (CTL2)	0.41	0.49
	Q03717	Potassium voltage-gated channel subfamily B member 1 (KCNB1)	1.31	1.25
	Q6PAM0	5’-AMP-activated protein kinase subunit beta-2 (PRKAB2)	0.49	0.45
	Q99J36	THUMP domain-containing protein 1 (THUMPD1)	1.55	1.60

## Data Availability

The proteomic data sets generated for this study are provided in the Supplementary Materials; further enquiries can be directed to the corresponding author.

## Supplementary Materials

The following supporting information can be downloaded at: https://www.mdpi.com/article/10.3390/antiox11050999/s1, Figure S1: Y-maze of 6-month-old WT and SELENOW KO mice.; Figure S2: Morris water maze of 6-month-old WT and SELENOW KO mice.; Figure S3: Nissl staining of the 6-month-old WT and SELENOW KO mice hippocampus.; Tables S1–S6: The detailed information including Uniprot ID, full name, fold change of DEPs from the TMT proteomic results.
