# Supplementary material for: Comparative Proteomic Analysis Reveals the Effect of Selenoprotein W Deficiency on Oligodendrogenesis in Fear Memory
Source: Antioxidants (Basel). 2022 May 19;11(5):999. doi: 10.3390/antiox11050999 (PMC9138053; doi:10.3390/antiox11050999)
Supplement: Supplementary file 1 [file antioxidants-11-00999-s001.zip › antioxidants-1700330-supplementary.pdf]

antioxidants

Supplementary Materials: Comparative Proteomic Analysis Reveals the Effect of Selenoprotein W Deficiency on Oligodendrogenesis in Fear Memory

Jiaxin Situ [1], Xuelian Huang [4], Mingyang Zuo [1], Yingying Huang [4], Bingyu Ren [1,2,*] and Qiong Liu [1,3,4,*]

[1] Shenzhen Key Laboratory of Marine Biotechnology and Ecology, College of Life Sciences and Oceanography, Shenzhen University, Shenzhen 518000, China; 2060251007@email.szu.edu.cn (J.S.); 2019301002@email.szu.edu.cn (M.Z.)
[2] Shenzhen-Hong Kong Institute of Brain Science-Shenzhen Fundamental Research Institutions, Shenzhen 518000, China
[3] Shenzhen Bay Laboratory, Shenzhen 518000, China
[4] Shenzhen Key Laboratory of Microbial Genetic Engineering, College of Life Sciences and Oceanography, Shenzhen University, Shenzhen 518000, China; 2100251006@email.szu.edu.cn (X.H.); 2020302013@email.szu.edu.cn (Y.H.)
* Correspondence: renbingyu@szu.edu.cn (B.R.); liuqiong@szu.edu.cn (Q.L.)

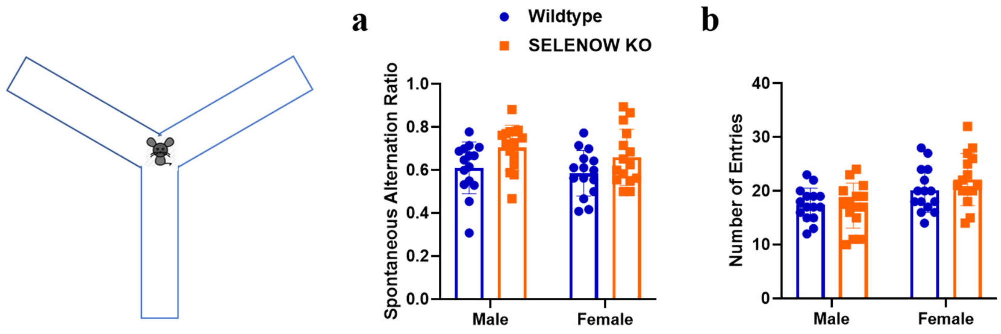

Figure S1. Y-maze of 6-month-old WT and SELENOW KO mice. Spatial memory of the 6-month-old WT and KO mice evaluated by the (**a**) spontaneous alternation ratio [defined as Nalt /(Ntotal-2); Nalt: number of alternations, an alternation is achieved when an animal enters a new arm rather than returning to one visited previously. Ntotal: number of total arm entries] and (**b**) number of total arm entries in the Y-maze task.

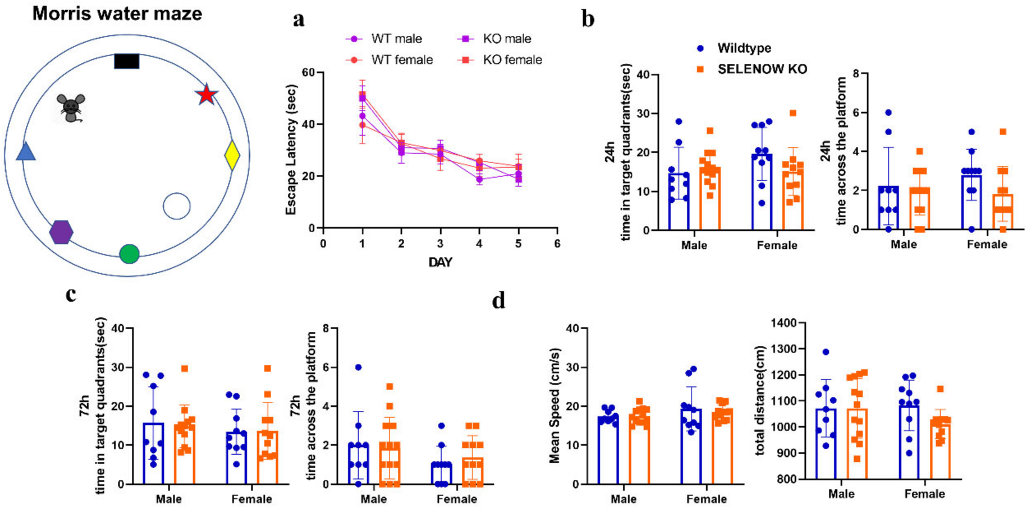

Figure S2. Morris water maze of 6-month-old WT and SELENOW KO mice. A. In a 5-day trial, the escape latency was recorded to evaluate the spatial memory and learning ability of the mice. No significant statistical difference had been found between WT and KO. B. The time of the mice spending in the target quadrant (left) and the number of times the mice crossing the platform (right) were recorded 24 h after the 5-day trial. No significant statistical difference had been found between WT and KO. C.The time of the mice spending in the target quadrant (left) and the number of times the mice crossing the platform (right) were recorded 72 h after the 5-day trial. No significant statistical difference had been found between WT and KO. D. The mean speed (left) and total distance (right) were recorded 72 h after the 5-day trial. No significant statistical difference had been found between WT and KO.

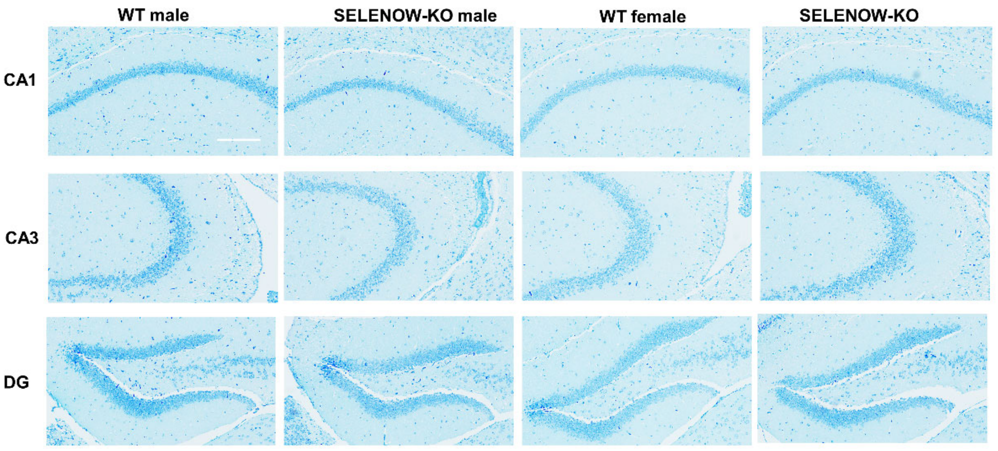

Figure S3. Nissl staining of the 6-month-old WT and SELENOW KO mice hippocampus. The Nissl staining results of hippocampus CA1 (upper panel), CA3 (middle panel) and DG (lower panel) regions of the 6-month-old NC and SELENOW KO mice. No abnormal Nissl bodies and morphological changes of hippocampus were found in each group. Scale bar = 200μm.

Table S1. The detailed information including Uniprot ID, full name, fold change above DEPs detected between KO NS and WT NS in hippocampus.

Uniprot ID	Name	Fold change
F7A0B0	Myelin basic protein	1.93
A0A0A6YXW6	Immunoglobulin heavy constant alpha	1.65
Q921C1	Gap junction gamma-3 protein	1.58
P60202	Myelin proteolipid protein	1.57
P0C0S6	Histone H2A.Z	1.55
F6TYB7	Myelin basic protein	1.55
Q9QWL7	Keratin, type I cytoskeletal 17	1.50
Q60771	Claudin-11	1.45
Q80YN3	Breast carcinoma-amplified sequence 1 homolog	1.44
Q922J6	Tetraspanin-2	1.42
P16330	2',3'-cyclic-nucleotide 3'-phosphodiesterase	1.42
F6RT34	Myelin basic protein	1.41
Q9DC53	Copine-8	1.40
E9Q7M2	TSC22 domain family, member 2	1.40
Q8K298	Anillin	1.39
C0HKE6	Histone H2A type 1-I	1.38
P40237	CD82 antigen	1.37
O09117	Synaptophysin-like protein 1	1.37
F6ZGN3	Unconventional myosin-XVIIIa (Fragment)	1.37
Q3UY21	Myelin-oligodendrocyte glycoprotein	1.35
G3UXY9	Ectonucleotide pyrophosphatase/phosphodiesterase family member 2	1.34
B2RY04	Dedicator of cytokinesis protein 5	1.34
Q9D2P8	Myelin-associated oligodendrocyte basic protein	1.34
Q5SYD0	Unconventional myosin-Id	1.31
Q3V0K9	Plastin-1	1.31
A0A087WPR1	Myelin-associated glycoprotein	1.31
Q8VDQ8	NAD-dependent protein deacetylase sirtuin-2	1.31
Q3UIA2	Rho GTPase-activating protein 17	1.31
P58742	Aladin	1.30
Q7M729	Sodium channel subunit beta-4	1.30
P84096	Rho-related GTP-binding protein RhoG	1.29
Q8R3P0	Aspartoacylase	1.29
Q924K8	Metastasis-associated protein MTA3	1.28
Q8K406	Leucine-rich repeat LGI family member 3	1.27
P63089	Pleiotrophin	1.27
F6SZ47	Serine/threonine-protein kinase B-raf (Fragment)	1.27
O35393	Ephrin-B3	1.27

P97315	Cysteine and glycine-rich protein 1	1.26
P24549	Retinal dehydrogenase 1	1.26
E9QMN8	Solute carrier family 15 member 2	1.26
O35474	EGF-like repeat and discoidin I-like domain-containing protein 3	1.26
Q9D154	Leukocyte elastase inhibitor A	1.25
Q5DU14	Unconventional myosin-XVI	1.25
Q60823	RAC-beta serine/threonine-protein kinase	1.25
Q8R310	Transmembrane and coiled-coil domain protein 3	1.25
P43276	Histone H1.5	1.25
Q6P8J2	Diamine acetyltransferase 2	1.25
Q91W61	F-box/LRR-repeat protein 15	1.25
Q9ESM3	Hyaluronan and proteoglycan link protein 2	1.25
P62320	Small nuclear ribonucleoprotein Sm D3	1.24
Q8BMQ8	Vacuolar fusion protein MON1 homolog B	1.24
Q9WV54	Acid ceramidase	1.24
Q9QYB1	Chloride intracellular channel protein 4	1.24
O88444	Adenylate cyclase type 1	1.24
Q80X85	28S ribosomal protein S7, mitochondrial	1.24
Q99L43	Phosphatidate cytidylyltransferase	1.24
Q80TG9	Leucine-rich repeat and fibronectin type-III domain-containing protein 2	1.24
A0A1W2P768	Histone H3.2	1.23
P50172	Corticosteroid 11-beta-dehydrogenase isozyme 1	1.23
B1AU74	Motile sperm domain-containing protein 2	1.23
Q64676	2-hydroxyacylsphingosine 1-beta-galactosyltransferase	1.23
Q9D071	MMS19 nucleotide excision repair protein homolog	1.22
P28867	Protein kinase C delta type	1.22
Q8K2T1	NmrA-like family domain-containing protein 1	1.22
G5E8G5	Prominin 1, isoform CRA_a	1.22
Q3V3R4	Integrin alpha-1	1.22
Q8BR26	Interferon-induced transmembrane protein 10	1.22
Q811C2	Cysteine protease ATG4C	1.22
Q61743	ATP-sensitive inward rectifier potassium channel 11	1.22
P47708	Rabphilin-3A	1.21
Q9WVS7	Dual specificity mitogen-activated protein kinase kinase 5	1.21
Q08642	Protein-arginine deiminase type-2	1.21
E9Q557	Desmoplakin	1.21
P51910	Apolipoprotein D	1.20

Q8K013	GTP-binding protein 10	1.20
P15864	Histone H1.2	1.20
Q09143	High affinity cationic amino acid transporter 1	1.20
Q61337	Bcl2-associated agonist of cell death	0.83
O88986	2-amino-3-ketobutyrate coenzyme A ligase, mitochondrial	0.83
Q8BXA0	Leucine-rich repeat and fibronectin type-III domain-containing protein 5	0.83
Q9QYM9	Tomoregulin-2	0.83
Q91VB8	Alpha globin 1	0.83
Q7TSZ8	Nucleus accumbens-associated protein 1	0.82
E9PZM8	Neuronal pentraxin receptor	0.82
A0A1W2P7Y9	Neuronal cell adhesion molecule	0.82
A0A075B6A0	Ig mu chain C region (Fragment)	0.81
Q8CDN6	Thioredoxin-like protein 1	0.81
Q505B7	Protein archease	0.81
O70340	Neuronal pentraxin-2	0.80
A0A0R4J000	BTB/POZ domain-containing protein KCTD5	0.80
Q9DAI2	Intraflagellar transport protein 22 homolog	0.79
Q9D7I5	Phospholysine phosphohistidine inorganic pyrophosphate phosphatase	0.79
A0A0G2JFV5	Leucine-rich repeat flightless-interacting protein 2	0.79
A0A1W2P6Z1	Neuronal cell adhesion molecule (Fragment)	0.79
H3BLD9	Zinc finger protein 428	0.79
Q9CR41	Huntingtin-interacting protein K	0.78
A2A654	Bromodomain PHD finger transcription factor	0.78
Q3UX10	Tubulin alpha chain-like 3	0.77
A6MDD2	Protein tyrosine phosphatase receptor type N	0.76
P01029	Complement C4-B	0.76
Q8R2H9	Phosphoethanolamine/phosphocholine phosphatase	0.76
Q6S9I3	HMW kininogen-II	0.75
A0A087WSF5	Leucine-rich repeat flightless-interacting protein 1 (Fragment)	0.75
Q3UNH4	G protein-regulated inducer of neurite outgrowth 1	0.75
P03987	Ig gamma-3 chain C region	0.73
Q9CQ89	Protein CutA	0.70
Q9R020	Zinc finger Ran-binding domain-containing protein 2	0.70
Q8BX80	Cytosolic endo-beta-N-acetylglucosaminidase	0.69
Q0VGU4	MCG18019	0.68
P20357	Microtubule-associated protein 2	0.68

Q9JJU8	SH3 domain-binding glutamic acid-rich-like protein	0.61
P63300	Selenoprotein W	0.53

Table S2. The detailed information including Uniprot ID, full name, fold change above DEPs detected between WT FC and WT NS in hippocampus.

**Uniprot ID**	**Name**	**Fold change**
F7A0B0	Myelin basic protein (Fragment)	1.76
Q921C1	Gap junction gamma-3 protein	1.60
Q60771	Claudin-11	1.43
Q9DC53	Copine-8	1.41
P21956	Lactadherin	1.41
Q922J6	Tetraspanin-2	1.40
F6TYB7	Myelin basic protein (Fragment)	1.39
Q8VI59	Pecanex-like protein 3	1.39
Q8K298	Anillin	1.38
Q3UY21	Myelin-oligodendrocyte glycoprotein	1.37
P16330	2',3'-cyclic-nucleotide 3'-phosphodiesterase	1.37
B2RY04	Dedicator of cytokinesis protein 5	1.36
Q3UIA2	Rho GTPase-activating protein 17	1.34
O88492	Perilipin-4	1.33
Q9D2P8	Myelin-associated oligodendrocyte basic protein	1.33
F6RT34	Myelin basic protein (Fragment)	1.33
P0C0S6	Histone H2A.Z	1.32
P40237	CD82 antigen	1.31
Q8R0J2	Mannose-P-dolichol utilization defect 1	1.30
Q924Z4	Ceramide synthase 2	1.30
Q9QWL7	Keratin, type I cytoskeletal 17	1.30
A0A0R4J0A6	Solute carrier family 17 (Sodium-dependent inorganic phosphate cotransporter), member 6, isoform CRA_a	1.29
Q8VDQ8	NAD-dependent protein deacetylase sirtuin-2	1.29
Q3V0K9	Plastin-1	1.28
Q8R3P0	Aspartoacylase	1.27
Q9D154	Leukocyte elastase inhibitor A	1.26
P84096	Rho-related GTP-binding protein RhoG	1.26
Q5SYD0	Unconventional myosin-Id	1.26
F6VQ81	Tumor protein D54 (Fragment)	1.26
O09117	Synaptophysin-like protein 1	1.26
Q99L88	Beta-1-syntrophin	1.26

Q8R310	Transmembrane and coiled-coil domain protein 3	1.25
Q924K8	Metastasis-associated protein MTA3	1.25
Q9ESN4	Complement C1q-like protein 3	1.25
Q9ESM3	Hyaluronan and proteoglycan link protein 2	1.24
Q1LZI2	Putative thiamine transporter SLC35F3	1.24
Q9QYB1	Chloride intracellular channel protein 4	1.24
Q8BHA3	Probable D-tyrosyl-tRNA(Tyr) deacylase 2	1.24
Q8K406	Leucine-rich repeat LGI family member 3	1.23
A0A087WPR1	Myelin-associated glycoprotein	1.23
A2A9Z2	Dystrophin	1.22
O35393	Ephrin-B3	1.22
P97315	Cysteine and glycine-rich protein 1	1.22
Q64310	Surfeit locus protein 4	1.21
Q9WVS7	Dual specificity mitogen-activated protein kinase kinase 5	1.21
Q99L43	Phosphatidate cytidylyltransferase 2	1.21
P02535	Keratin, type I cytoskeletal 10	1.21
G5E8G5	Prominin 1, isoform CRA_a	1.21
C0HKE6	Histone H2A type 1-I	1.21
Q9Z0S1	3'(2'),5'-bisphosphate nucleotidase 1	1.21
Q922U2	Keratin, type II cytoskeletal 5 OS=Mus musculus GN=Krt5 PE=1 SV=1	1.20
Q91W61	F-box/LRR-repeat protein 15	1.20
A0A1L1SQK2	Probable leucine--tRNA ligase, mitochondrial (Fragment)	1.20
Q6R891	Neurabin-2	0.83
Q9CRA5	Golgi phosphoprotein 3	0.83
Q3V0I2	Proline-rich protein 7	0.83
Q3UHT7	ERC protein 2	0.82
G3UW94	MCG14259, isoform CRA_b	0.82
A0A0U1RPL0	Ataxin-2-like protein	0.82
P23953	Carboxylesterase 1C	0.81
Q3TRM4	Neuropathy target esterase	0.81
Q9JKF7	39S ribosomal protein L39, mitochondrial	0.81
Q60864	Stress-induced-phosphoprotein 1	0.81
Q8BKZ9	Pyruvate dehydrogenase protein X component, mitochondrial	0.80
Q3UZG4	Aminoacyl tRNA synthase complex-interacting multifunctional protein 1	0.80
A0A075B6A0	Ig mu chain C region (Fragment)	0.80
A0A075B5P2	Immunoglobulin kappa constant (Fragment)	0.79
P38060	Hydroxymethylglutaryl-CoA lyase, mitochondrial	0.79

A6MDD2	Protein tyrosine phosphatase receptor type N	0.78
P01029	Complement C4-B	0.77
A0A087WSF5	Leucine-rich repeat flightless-interacting protein 1 (Fragment)	0.77
Q80X50	Ubiquitin-associated protein 2-like	0.76
Q61337	Bcl2-associated agonist of cell death	0.75
P97298	Pigment epithelium-derived factor	0.73
P40936	Indolethylamine N-methyltransferase	0.73
Q8BX80	Cytosolic endo-beta-N-acetylglucosaminidase	0.68
Q9QXT0	Protein canopy homolog 2	0.60
Q3UV17	Keratin, type II cytoskeletal 2 oral	0.59

Table S3. The detailed information including Uniprot ID, full name, fold change above DEPs detected between KO FC and KO NS in hippocampus.

**Uniprot ID**	**Name**	**Fold change**
Q6P9L6	Kinesin-like protein KIF15	1.69
P59108	Copine-2	1.37
Q0VE82	Copine-7	1.35
Q501J7	Phosphatase and actin regulator 4	1.29
Q8BG58	Transmembrane prolyl 4-hydroxylase	1.28
Q9WTS4	Teneurin-1	1.27
A2A654	Bromodomain PHD finger transcription factor	1.27
Q9DB30	Phosphorylase b kinase gamma catalytic chain, liver/testis isoform	1.26
Q6P5E6	ADP-ribosylation factor-binding protein GGA2	1.26
Q9JJZ2	Tubulin alpha-8 chain	1.25
Q8C0D0	Probable tRNA pseudouridine synthase 1	1.25
Q8R2H9	Phosphoethanolamine/phosphocholine phosphatase	1.24
E9QP54	Jouberin O	1.22
Q6IR34	G-protein-signaling modulator 1	1.22
O88986	2-amino-3-ketobutyrate coenzyme A ligase, mitochondrial	1.22
O70401	Tetraspanin-6	1.22
Q9WTR5	Cadherin-13	1.21
Q8C4Q6	Axin interactor, dorsalization-associated protein	1.21
Q80Z24	Neuronal growth regulator 1	1.21
Q923L3	CUB and sushi domain-containing protein 1	1.20
O89084	cAMP-specific 3',5'-cyclic phosphodiesterase 4A	0.83
P24549	Retinal dehydrogenase 1	0.83

G3XA00	Glutamate receptor, metabotropic 4	0.83
O09117	Synaptophysin-like protein 1	0.83
Q8QZV7	Integrator complex subunit 13	0.83
E9PX57	Kinesin-like protein	0.82
P50172	Corticosteroid 11-beta-dehydrogenase isozyme 1	0.82
E9PYV1	Glucocorticoid receptor	0.81
F6ZDE5	Rap guanine nucleotide exchange factor (GEF) 1 (Fragment)	0.81
Q7M729	Sodium channel subunit beta-4	0.80
Q8BUN5	Mothers against decapentaplegic homolog 3	0.79
D3Z4E2	Neuritin	0.79
G3UXY9	Ectonucleotide pyrophosphatase/phosphodiesterase family member 2	0.77
P22599	Alpha-1-antitrypsin 1-2	0.74
Q04859	Serine/threonine-protein kinase MAK	0.69
P0C7L0	WAS/WASL-interacting protein family member 3	0.68
Q8K4P8	E3 ubiquitin-protein ligase HECW1	0.58

Table S4. The detailed information including Uniprot ID, full name, fold change above DEPs detected between KO NS and WT NS in Amygdala.

Uniprot ID	Name	Fold change
B1AVF5	Probable E3 ubiquitin-protein ligase MID2	1.73
P84104	Serine/arginine-rich splicing factor 3	1.56
A0A1L1SQ79	Pleckstrin homology domain-containing family A member 7 (Fragment)	1.54
NP_001039526.1	cytochrome c [Bos taurus]	1.47
Q99J95	Cyclin-dependent kinase 9	1.47
P03930	ATP synthase protein 8	1.46
Q9D7N3	28S ribosomal protein S9, mitochondrial	1.46
P98191	Phosphatidate cytidylyltransferase 1	1.42
A2AQJ8	Neutral alpha-glucosidase C	1.40
C0HKE6	Histone H2A type 1-I	1.38
E9QPX1	Collagen alpha-1(XVIII) chain	1.36
P63030	Mitochondrial pyruvate carrier 1	1.35
Q99N91	39S ribosomal protein L34, mitochondrial	1.35
Q9D1B9	39S ribosomal protein L28, mitochondrial	1.34
Q8VE95	UPF0598 protein C8orf82 homolog	1.34
A0A0A6YWR2	Ig gamma-1 chain C region secreted form (Fragment)	1.32

Q8VE73	Cullin-7	1.32
Q9JJF3	Ribosomal oxygenase 1	1.31
Q3TC33	Coiled-coil domain-containing protein 127	1.31
P40630	Transcription factor A, mitochondrial	1.30
Q06185	ATP synthase subunit e, mitochondrial	1.30
Q8VC82	Rhomboid-related protein 1	1.30
Q8K215	LYR motif-containing protein 4	1.29
Q31125	Zinc transporter SLC39A7	1.28
Q9CQX8	28S ribosomal protein S36, mitochondrial	1.28
Q6P9Z1	SWI/SNF-related matrix-associated actin-dependent regulator of chromatin subfamily D member 3	1.28
Q9CYW4	Haloacid dehalogenase-like hydrolase domain-containing protein 3	1.27
Q9CQC7	NADH dehydrogenase [ubiquinone] 1 beta subcomplex subunit 4	1.27
Q9CPQ8	ATP synthase subunit g, mitochondrial	1.27
P27661	Histone H2AX	1.26
Q8VE22	28S ribosomal protein S23, mitochondrial	1.25
Q9R013	Cathepsin F	1.25
F6U3S2	Major facilitator superfamily domain-containing protein 6 (Fragment)	1.25
Q8VEH8	Endoplasmic reticulum lectin 1	1.25
P63137	Gamma-aminobutyric acid receptor subunit beta-2	1.24
Q149F3	Eukaryotic peptide chain release factor GTP-binding subunit ERF3B	1.24
P49070	Calcium signal-modulating cyclophilin ligand	1.23
Q8VDU5	SNF-related serine/threonine-protein kinase	1.23
Q8K449	ATP-binding cassette sub-family A member 9	1.23
A0A0R4J0D1	Store-operated calcium entry-associated regulatory factor	1.22
Q91VR2	ATP synthase subunit gamma, mitochondrial	1.20
P62137	Serine/threonine-protein phosphatase PP1-alpha catalytic subunit	1.20
D3Z2L2	Rab5 GDP/GTP exchange factor (Fragment)	1.20
P50428	Arylsulfatase A	0.83
E9PZD8	Ceruloplasmin	0.83
Q3TTF2	Putative uncharacterized protein	0.82
Q6Y7W8	GRB10-interacting GYF protein 2	0.82
A0A1D5RLT6	GRAM domain-containing protein 1B	0.81
Q3TCH7	Cullin-4A	0.81

P48774	Glutathione S-transferase Mu 5	0.81
A0A087WNU5	Ankyrin-3 (Fragment)	0.81
Q8K3W3	Protein CASC3	0.81
Q69Z98	Serine/threonine-protein kinase BRSK2	0.81
Q8BJW6	Eukaryotic translation initiation factor 2A	0.80
Q61481	Calcium/calmodulin-dependent 3',5'-cyclic nucleotide phosphodiesterase 1A	0.79
Q9JMH7	Sialidase-3	0.78
Q8VEK2	Rhomboid domain-containing protein 2	0.77
Q52KI8	Serine/arginine repetitive matrix protein 1	0.77
Q91Z25	Actin-related protein 2/3 complex subunit 1B	0.77
Q6ZWQ7	Signal peptidase complex subunit 3	0.77
A2AR50	Ras-specific guanine nucleotide-releasing factor RalGPS1	0.77
P27546	Microtubule-associated protein 4	0.77
S4R1Y1	Cullin-9	0.76
S4R2R5	Ankyrin-2	0.75
A2A5V4	SH3 domain-binding protein 1	0.74
Q8BGP6	Solute carrier family 25 member 40	0.74
G5E8C4	MCG142017, isoform CRA_a	0.73
Q3V4B5	COMM domain-containing protein 6	0.73
Q9R1Z7	6-pyruvoyl tetrahydrobiopterin synthase	0.72
Q8CDA1	Phosphatidylinositide phosphatase SAC2	0.70
P09405	Nucleolin	0.69
A0A075B6A0	Ig mu chain C region (Fragment)	0.68
A2ATU0	Probable 2-oxoglutarate dehydrogenase E1 component DHKTD1, mitochondrial	0.66
P03987	Ig gamma-3 chain C region	0.64
F6TQW2	Immunoglobulin heavy constant gamma 2C	0.62
Q9QZA0	Carbonic anhydrase 5B, mitochondrial	0.62
P29391	Ferritin light chain 1	0.60
Q3UHX2	28 kDa heat- and acid-stable phosphoprotein	0.58
Q80ZI6	E3 ubiquitin-protein ligase LRSAM1	0.55
P09528	Ferritin heavy chain	0.44
B7ZCQ3	MCG12939, isoform CRA_c	0.31
P02104	Hemoglobin subunit epsilon-Y2	0.22

Table S5. The detailed information including Uniprot ID, full name, fold change above DEPs detected between WT FC and WT NS in Amygdala.

Uniprot ID	Name	Fold change
Q91YN1	Protein FAM118A	2.16
Q80Y14	Glutaredoxin-related protein 5, mitochondrial	1.96
E9PUE1	R3H and coiled-coil domain-containing protein 1	1.62
B1AVF5	Probable E3 ubiquitin-protein ligase MID2	1.61
Q99J36	THUMP domain-containing protein 1	1.55
P97291	Cadherin-8	1.47
E9PVE8	Pituitary adenylate cyclase-activating polypeptide type I receptor	1.47
P60840	Alpha-endosulfine	1.46
Q9R0L7	A-kinase anchor protein 8-like	1.45
P12961	Neuroendocrine protein 7B2	1.44
Q99J95	Cyclin-dependent kinase 9	1.44
Q924K8	Metastasis-associated protein MTA3	1.43
Q3UVU8	Disks large-associated protein 1	1.40
Q8VED9	Galectin-related protein	1.39
Q9EQ41	Vomeronasal type-1 receptor	1.39
Q99N91	39S ribosomal protein L34, mitochondrial	1.35
P0DI97	Neurexin-1-beta	1.35
Q9R0U0	Serine/arginine-rich splicing factor 10	1.33
G3UW70	MCG21719	1.33
Q03717	Potassium voltage-gated channel subfamily B member 1	1.31
P07310	Creatine kinase M-type	1.31
E9Q0V6	NHS-like protein 2	1.31
Q8VC82	Rhomboid-related protein 1	1.30
J3QK20	Voltage-dependent L-type calcium channel subunit beta-4	1.29
Q9DAI2	Intraflagellar transport protein 22 homolog	1.29
P03930	ATP synthase protein 8	1.28
Q80YQ8	Protein RMD5 homolog A	1.28
P51163	Uroporphyrinogen-III synthase	1.27
P60764	Ras-related C3 botulinum toxin substrate 3	1.27
E9PXF0	Protocadherin 17	1.27
Q6P2L7	Protein CASC4	1.27
Q8BG16	Sodium-dependent neutral amino acid transporter B(0)AT2	1.26
E9QP00	Transformer-2 protein homolog alpha	1.25
Q9CT10	Ran-binding protein 3	1.25
P62843	40S ribosomal protein S15	1.25

Q9JIK9	28S ribosomal protein S34, mitochondrial	1.25
D3Z7Q5	Programmed cell death protein 5	1.25
Q3TUQ5	Pinin	1.23
Q6PDI6	Ubiquitin carboxyl-terminal hydrolase MINDY-2	1.23
Q8BGC0	HIV Tat-specific factor 1 homolog	1.22
Q8BHT6	Beta-1,3-glucosyltransferase	1.22
Q8C460	ERI1 exoribonuclease 3	1.21
Q9JIZ9	Phospholipid scramblase 3	1.21
Q9R013	Cathepsin F	1.21
Q99K10	Neuroligin-1	1.21
Q924Z6	Exportin-6	1.20
P63137	Gamma-aminobutyric acid receptor subunit beta-2	1.20
Q8VDS4	Regulation of nuclear pre-mRNA domain-containing protein 1A	1.20
O88738	Baculoviral IAP repeat-containing protein 6	0.83
Q8VEK2	Rhomboid domain-containing protein 2	0.83
A0A1W2P6J7	Septin 10	0.83
A2APT9	Kelch domain-containing protein 7A	0.83
P26041	Moesin	0.83
D6RH77	Phospholipase D1	0.83
Q8BGS7	Choline/ethanolaminephosphotransferase 1	0.83
Q8BZW8	NHL repeat-containing protein 2	0.83
Q6P5D3	Putative ATP-dependent RNA helicase DHX57	0.83
F6Q8D3	Phospholipid-transporting ATPase	0.82
Q8R1X6	Spartin	0.82
P26043	Radixin	0.82
Q9DBN5	Lon protease homolog 2, peroxisomal	0.82
O35393	Ephrin-B3	0.82
P17047	Lysosome-associated membrane glycoprotein 2 OS=Mus musculus GN=Lamp2 PE=1 SV=2	0.82
G3X9G4	Dynamin-2	0.81
O08644	Ephrin type-B receptor 6	0.81
B1ATI0	Aldehyde dehydrogenase	0.81
Q8BMD6	Transmembrane protein 260	0.81
E9PZD8	Ceruloplasmin	0.81
A2A5V4	SH3 domain-binding protein 1	0.80
Q8VCU2	Glycosylphosphatidylinositol specific phospholipase D1	0.80
P19324	Serpin H1	0.80
Q8BG51	Mitochondrial Rho GTPase 1	0.80
Q9D666	SUN domain-containing protein 1	0.79

Q3UDF0	Putative uncharacterized protein	0.79
Q8VE11	Myotubularin-related protein 6	0.79
Q8BJW6	Eukaryotic translation initiation factor 2A	0.78
Q9D964	Glycine amidinotransferase, mitochondrial	0.78
G3X9Y9	Myosin Vb, isoform CRA_a	0.78
O88533	Aromatic-L-amino-acid decarboxylase	0.78
Q9JMH7	Sialidase-3	0.78
A0A0R4J0S4	Lethal(2) giant larvae protein homolog 1	0.78
Q9CRC0	Vitamin K epoxide reductase complex subunit 1	0.77
Q3U7M5	Protein ABHD4	0.77
Q9QXX4	Calcium-binding mitochondrial carrier protein Aralar2	0.77
P13020	Gelsolin	0.77
Q8BH82	N-acyl-phosphatidylethanolamine-hydrolyzing phospholipase D	0.77
Q61247	Alpha-2-antiplasmin OS=Mus musculus GN=Serpinf2 PE=1 SV=1	0.77
P01897	H-2 class I histocompatibility antigen, L-D alpha chain	0.76
Q06770	Corticosteroid-binding globulin	0.76
Q9JHF5	V-type proton ATPase subunit a	0.75
Q8BGP6	Solute carrier family 25 member 40	0.75
E9Q035	Uncharacterized protein	0.75
Q91XY6	MCG133388, isoform CRA_c	0.75
A0A0G2JEU7	Immunoglobulin heavy variable 1-82 (Fragment)	0.75
Q8CDA1	Phosphatidylinositide phosphatase SAC2	0.75
Q64669	NAD(P)H dehydrogenase [quinone] 1	0.75
A0A0A6YW53	Mitogen-activated protein kinase kinase kinase kinase 4	0.74
A0A0R4J0I1	MCG1051009 OS=Mus musculus	0.74
P32211	Muscarinic acetylcholine receptor M4	0.74
P11438	Lysosome-associated membrane glycoprotein 1 OS=Mus musculus GN=Lamp1 PE=1 SV=2	0.74
Q921I1	Serotransferrin	0.74
P01029	Complement C4-B	0.73
Q9CY21	Probable 18S rRNA (guanine-N(7))-methyltransferase	0.73
Q3V4B5	COMM domain-containing protein 6	0.73
Q9QY39	PDZ domain-containing protein 4	0.72
Q8BYJ6	TBC1 domain family member 4	0.72
E9Q4K7	Kinesin family member 13B	0.72
Q91X72	Hemopexin	0.71

A0A075B5P3	Immunoglobulin heavy constant gamma 2B (Fragment)	0.71
P22599	Alpha-1-antitrypsin 1-2	0.70
Q91VC9	Growth hormone-inducible transmembrane protein	0.70
E9Q8I0	Complement factor H	0.70
P07724	Serum albumin OS=Mus musculus GN=Alb PE=1 SV=3	0.70
P23953	Carboxylesterase 1C	0.70
E0CXZ8	Dynamin-3	0.70
P29391	Ferritin light chain 1	0.69
G5E8C4	MCG142017, isoform CRA_a	0.69
A0A075B6A0	Ig mu chain C region (Fragment)	0.66
E9QM38	Solute carrier family 12 member 2	0.65
A0A140LJ69	Protein unc-13 homolog B	0.65
E9Q3X0	Major vault protein	0.65
P61979	Heterogeneous nuclear ribonucleoprotein K	0.62
F7DBB3	AHNAK nucleoprotein 2 (Fragment)	0.62
B2RXE2	Sodium/hydrogen exchanger	0.61
Q8BGN3	Ectonucleotide pyrophosphatase/phosphodiesterase family member 6	0.60
Q80ZI6	E3 ubiquitin-protein ligase LRSAM1	0.58
A2ATU0	Probable 2-oxoglutarate dehydrogenase E1 component DHKTD1, mitochondrial	0.55
Q924Z4	Ceramide synthase 2	0.55
F6TQW2	Immunoglobulin heavy constant gamma 2C	0.53
Q6PAM0	5'-AMP-activated protein kinase subunit beta-2	0.49
P09528	Ferritin heavy chain	0.47
A0A1L1SU40	Choline transporter-like protein 2 (Fragment)	0.41
Q61327	Sodium-dependent dopamine transporter	0.36
P02104	Hemoglobin subunit epsilon-Y2	0.20

Table S6. The detailed information including Uniprot ID, full name, fold change above DEPs detected between KO FC and KO NS in Amygdala.

Uniprot ID	Name	Fold change
Q99J36	THUMP domain-containing protein 1	1.60
Q924T7	E3 ubiquitin-protein ligase RNF31	1.59
Q69ZL1	FYVE, RhoGEF and PH domain-containing protein 6	1.47
S4R2R5	Ankyrin-2	1.40
Q03391	Glutamate receptor ionotropic, NMDA 2D	1.37
A2ATS6	Reticulon-4 receptor-like 2 (Fragment)	1.37
Q8K3W3	Protein CASC3	1.36
A2ARP8	Microtubule-associated protein 1A	1.32
A0A1D5RLT6	GRAM domain-containing protein 1B	1.31
Q52KI8	Serine/arginine repetitive matrix protein 1	1.29
A0A0U1RPM3	PRA1 family protein	1.27
P14873	Microtubule-associated protein 1B	1.26
P62960	Nuclease-sensitive element-binding protein 1	1.25
Q03717	Potassium voltage-gated channel subfamily B member 1	1.25
Q7TQE6	Macoilin O	1.24
A0A0R4J098	Zinc finger protein 326	1.24
Q9CYC6	m7GpppN-mRNA hydrolase O	1.22
Q5XKN4	Protein jagunal homolog 1	1.21
Q8VDZ4	Palmitoyltransferase ZDHHC5	1.20
Q8BTS4	Nuclear pore complex protein Nup54 O	0.83
Q9CR00	26S proteasome non-ATPase regulatory subunit 9	0.82
Q99M51	Cytoplasmic protein NCK1	0.82
Q921W0	Charged multivesicular body protein 1a	0.82
E9QN99	Protein ABHD14B	0.81
P34884	Macrophage migration inhibitory factor	0.81
O88983	Syntaxin-8	0.81
P20152	Vimentin	0.80
Q6P9N1	Hyccin	0.80
P35831	Tyrosine-protein phosphatase non-receptor type 12	0.80
Q9QYI5	DnaJ homolog subfamily B member 2	0.77
Q99PG2	Opioid growth factor receptor	0.76
Q9DCC7	Isochorismatase domain-containing protein 2B	0.76
D3Z0L4	MICOS complex subunit (Fragment)	0.75
E9QPX1	Collagen alpha-1(XVIII) chain	0.74
Q9D104	Putative uncharacterized protein	0.74

A0A1L1SQ79	Pleckstrin homology domain-containing family A member 7 (Fragment)	0.73
C0HKE6	Histone H2A type 1-I	0.73
E9QLZ1	TSC22 domain family protein 1	0.71
Q9D7N3	28S ribosomal protein S9, mitochondrial	0.70
Q8BX94	Oxysterol-binding protein-related protein 2	0.70
Q64433	10 kDa heat shock protein, mitochondrial	0.69
A0A075B6A0	Ig mu chain C region (Fragment)	0.69
P84104	Serine/arginine-rich splicing factor 3	0.67
NP_001039526.1	cytochrome c	0.67
Q99JR5	Tubulointerstitial nephritis antigen-like	0.55
A0A1L1SU40	Choline transporter-like protein 2 (Fragment)	0.49
Q6PAM0	5'-AMP-activated protein kinase subunit beta-2	0.45
